# *In Vivo* Validation of *In Silico* Predicted Metabolic Engineering Strategies in Yeast: Disruption of α-Ketoglutarate Dehydrogenase and Expression of ATP-Citrate Lyase for Terpenoid Production

**DOI:** 10.1371/journal.pone.0144981

**Published:** 2015-12-23

**Authors:** Evamaria Gruchattka, Oliver Kayser

**Affiliations:** Technical Biochemistry, Department of Biochemical and Chemical Engineering, TU Dortmund University, Emil-Figge-Str. 66, 44227, Dortmund, Germany; Universidad de La Laguna, SPAIN

## Abstract

**Background:**

Engineering of the central carbon metabolism of *Saccharomyces cerevisiae* to redirect metabolic flux towards cytosolic acetyl-CoA has become a central topic in yeast biotechnology. A cell factory with increased flux into acetyl-CoA can be used for heterologous production of terpenoids for pharmaceuticals, biofuels, fragrances, or other acetyl-CoA derived compounds. In a previous study, we identified promising metabolic engineering targets in *S*. *cerevisiae* using an *in silico* stoichiometric metabolic network analysis. Here, we validate selected *in silico* strategies *in vivo*.

**Results:**

Patchoulol was produced by yeast via a heterologous patchoulol synthase of *Pogostemon cablin*. To increase the metabolic flux from acetyl-CoA towards patchoulol, a truncated HMG-CoA reductase was overexpressed and farnesyl diphosphate synthase was fused with patchoulol synthase. The highest increase in production could be achieved by modifying the carbon source; sesquiterpenoid titer increased from glucose to ethanol by a factor of 8.4. Two strategies predicted *in silico* were chosen for validation in this work. Disruption of α-ketoglutarate dehydrogenase gene (*KGD1*) was predicted to redirect the metabolic flux via the pyruvate dehydrogenase bypass towards acetyl-CoA. The metabolic flux was redirected as predicted, however, the effect was dependent on cultivation conditions and the flux was interrupted at the level of acetate. High amounts of acetate were produced. As an alternative pathway to synthesize cytosolic acetyl-CoA, ATP-citrate lyase was expressed as a polycistronic construct, however, *in vivo* performance of the enzyme needs to be optimized to increase terpenoid production.

**Conclusions:**

Stoichiometric metabolic network analysis can be used successfully as a metabolic prediction tool. However, this study highlights that kinetics, regulation and cultivation conditions may interfere, resulting in poor *in vivo* performance. Main sites of regulation need to be released and improved enzymes are essential to meet the required activities for an increased product formation *in vivo*.

## Introduction

Terpenoids are one of the largest classes of natural products comprising of tens of thousands of compounds. These hydrocarbon compounds have multiple applications from pharmaceuticals to biofuels and fragrances [1]. Heterologous production of terpenoids with the yeast *Saccharomyces cerevisiae* has become popular due to advances in molecular biology tools and the general robustness of this organism in fermentation processes [2]. Metabolic engineering, which involves detailed metabolic analysis to identify targets, followed by directed genetic modifications for improvement of cells via recombinant DNA technology [3], represents a strategy to increase heterologous terpenoid formation from the yeast host. Early heterologous terpenoid production studies in yeast focused on engineering the native terpenoid biosynthetic pathway, the mevalonate pathway (MVA), by several strategies including: overexpressing a truncated and thus soluble as well as non-regulated version of HMG-CoA reductase (t*HMG1*) [4, 5], down-regulating squalene synthase (*ERG9*), which leads via several steps to the major end product ergosterol [6, 7] as well as overexpression of a mutant transcription factor *upc2-1* upregulating several MVA pathway genes [5]. However, little research had been done concerning modifications within the central carbon metabolism to redirect the metabolic flux towards terpenoids. Thus, our group has previously analyzed the metabolic networks of *S*. *cerevisiae* and also *Escherichia coli*, the most prominently used hosts for heterologous terpenoid production, and identified promising metabolic engineering strategies *in silico* using a stoichiometric metabolic network analysis [8]. Based on our analysis, both hosts have the potential to produce terpenoids to higher yields than previously achieved with these newly identified targets for metabolic engineering. *S*. *cerevisiae* was chosen here for this validation study due to its robustness and the higher probability to functionally express plant P450 enzymes, which are necessary for the biosynthesis of several terpenoids [9, 10]. Different carbon sources were analysed for their potential to supply terpenoids in high yields and ethanol was identified as the most promising carbon source for yeast [8]. However, ethanol is an expensive carbon substrate, and less expensive alternatives in the form of sugars are preferable. Molasses and hemicellulosic hydrolysates of agricultural by-products represent inexpensive carbon substrates which contain high amounts of simple sugars [11]. Metabolic engineering strategies identified *in silico*, which employ glucose as a substrate, can as well be applied to other sugars like fructose, galactose or even xylose [8]. Thus, the focus here was set on the *in vivo* validation of metabolic engineering strategies based on glucose as carbon source in the yeast *S*. *cerevisiae*. Patchoulol synthase (Pts), which produces the sesquiterpenoid patchoulol as its main product, was chosen as reporter in this study.

**Fig 1:**
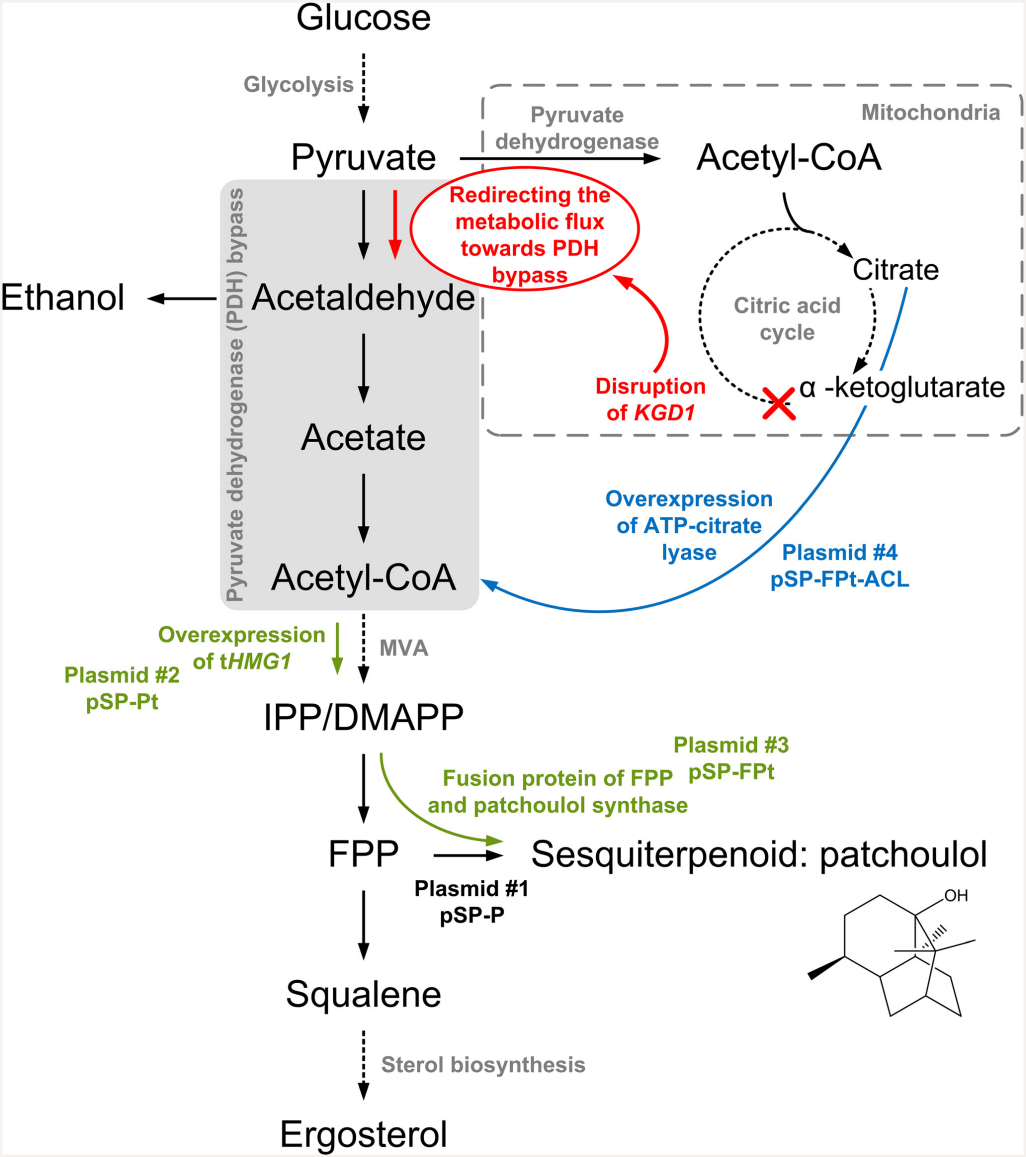
Metabolic engineering approaches in yeast for increased terpenoid production. A simplified metabolic network of yeast depicting most important reactions is shown. Production of the sesquiterpenoid patchoulol is enabled via expression of patchoulol synthase (via plasmid #1 pSP-P). Overexpression of truncated HMG-CoA reductase (t*HMG1*, via plasmid #2 pSP-Pt) and fusion of FPP synthase with patchoulol synthase (via plasmid #3 pSP-FPt) to increase the flux from the central carbon precursor acetyl-CoA to the terpenoid product are highlighted in green. The disruption of α-ketoglutarate dehydrogenase (*KGD1*) to redirect the metabolic flux from citric acid cycle via pyruvate dehydrogenase bypass towards terpenoids is shown in red. Overexpression of ATP-citrate lyase (via plasmid #4 pSP-FPt-ACL) to produce cytosolic acetyl-CoA via pyruvate dehydrogenase and citric acid cycle instead of pyruvate dehydrogenase bypass is shown in blue.

In this report, we analyze one metabolic engineering strategy *in vivo*, which was based on the constrained minimal cut sets approach [12]. In this approach a gene knockout within the citric acid cycle (α-ketoglutarate dehydrogenase) in addition to inhibiting ethanol and acetate production by the cell was predicted to yield high productivities of terpenoids. The rationale behind this strategy is that the metabolic flux is redirected from citric acid cycle via the pyruvate dehydrogenase bypass towards acetyl-CoA for increased terpenoid production (see Fig 1).

A second engineering target was a heterologous pathway to synthesize cytosolic acetyl-CoA. Pyruvate dehydrogenase bypass is the major source of cytosolic acetyl-CoA in *S*. *cerevisiae*. However, most other organisms possess ATP-citrate lyase as alternative pathway to synthesize cytosolic acetyl-CoA (see Fig 1). The heterologous enzyme was identified as promising metabolic engineering target for increased terpenoid production in our *in silico* computation [8]. The rationale behind this is that citrate is the substrate for acetyl-CoA synthesis and is produced via the pyruvate dehydrogenase complex and citric acid cycle, circumventing pyruvate dehydrogenase bypass and especially acetyl-CoA synthetase, which requires one additional mol ATP per acetyl-CoA.

This work was conducted in order to confirm these predictions with all discussed possibilities and limitations and identify possible considerations in order to improve stoichiometric metabolic network predictions *in silico* and reliably increase productivity *in vivo*. In addition, this study includes prerequisites important for validating metabolic engineering strategies within the central carbon metabolism for enhanced terpenoid production, *i*.*e*. a two-phase cultivation and identification of the product spectrum of the terpenoid synthase. Furthermore, the flux in the mevalonate pathway was increased towards the desired product via overexpression of a truncated HMG-CoA reductase (t*HMG1*) and fusion of FPP synthase with patchoulol synthase to ensure that a modified flux distribution in the central carbon metabolism is resulting in increased terpenoid formation.

## Material and Methods

### Strains, media and growth conditions

Plasmids were amplified in *E*. *coli* DH5α (LifeTechnologies). Cells were cultured at 37°C and 200 rpm in LB medium (5 g/L yeast extract, 10 g/L tryptone, 10 g/L NaCl, pH 7, for solid medium 20 g/L agar) containing 100 mg/L ampicillin when necessary. Yeast strain *S*. *cerevisiae* CEN.PK2-1C (*MAT* **a**; *ura3-52 MAL2-8C SUC2*; Euroscarf) was used as background in all experiments. Yeast strains were cultured at 30°C and 200 rpm in unbaffled shake flasks. Yeast strains not carrying a plasmid were grown in YM (11 g/L glucose x H_2_O, 3 g/L yeast extract, 5 g/L peptone and 3 g/L malt extract, for solid medium 20 g/L agar). Yeast strains carrying a plasmid were grown in synthetic dextrose (SD) medium containing 20 g/L glucose x H_2_O, 6.7 g/L yeast nitrogen base without amino acids, 10.2 g/L KH-phthalate, adjusted to pH 5.5 with KOH and for solid medium 20 g/L agar. Strains were stored in 30% glycerol at -80°C in cryo-vials.

### Plasmid construction and transformation

Plasmids used and constructed in this study are depicted in Table 1, maps of expression plasmids are shown in S10 Fig, gene sequences are shown in S1 Table and primers used in this study are shown in S2 Table. Plasmids were constructed exploiting yeasts homologous recombination machinery. Therefore fragments containing approx. 30–60 bp homologous overlap regions were amplified via PCR and transformed into yeast (250 ng per insert) together with the linearized target plasmid (500 ng). Yeast transformation was performed using a standard lithium acetate procedure [13]. Plasmids were isolated from yeast, transformed into *E*. *coli* for amplification using a standard procedure for chemically competent cells and heat shock transformation [14] and isolated for verification via PCR and sequencing. Phusion^®^ High-Fidelity DNA Polymerase (Finnzymes/Thermo Scientific) was used for amplifications, *Taq* DNA Polymerase 1.1x Master Mix Red (Ampliqon, biomol) was used to verify correct integrations.

**Table 1:** Plasmids used in this study.

Plasmid	Characteristics	Source
pUG6	Plasmid carrying loxP-flanked marker gene deletion cassette: loxP-P_TEF1_-kanMX-T_TEF1_-loxP	Euroscarf
pSH47	Plasmid carrying Cre recombinase for loxP mediated marker gene removal	Euroscarf
P423H7	Plasmid carrying truncated HXT7 promoter (-392 bp to -1 bp)	[15]
pYX042-ACLA-1	Plasmid carrying cDNA *ACLA-1*	[16]
pYX012-ACLB-2	Plasmid carrying cDNA *ACLB-2*	[16]
pSP-GM1	Yeast expression plasmid containing bidirectional promoters P_*TEF1*_ and P_*PGK1*_	[17]
pSP-P	pSP-GM1 carrying the sequence optimized patchoulol synthase cDNA (*PTS*) from *Pogostemon cablin* Benth.—(P_*TEF1*_ *-PTS*),	this study
pSP-Pt	pSP-P carrying the truncated (first 1590 bp are cut, start codon was reintroduced) HMG-Co reductase gene (t*HMG1*) from *S*. *cerevisiae* CEN.PK2-1C—(P_*TEF1*_ *-PTS*, P_*PGK1*_-t*HMG*)	this study
pSP-FPt	pSP-Pt carrying the farnesol diphosphate synthase gene (*ERG20*) from *S*. *cerevisiae* CEN.PK2-1C fused to PTS via a short flexible linker (GSG)—(P_*TEF1*_ *-FPPS-PTS*, P_*PGK1*_-t*HMG*)	this study
pSP-FPt-ACL	pSP-FPt carrying the ATP-citrate lyase cDNAs (*ACLA-1* and *ACLB-2*) from *Arabidopsis* [16] fused via T2A sequences from *Thosea asigna* virus—(P_*TEF1*_ *-FPPS-PTS*, P_*PGK1*_-t*HMG*, P_HXT7*_-*ACLA-1*-T2A-*ACLB-2*)	this study

The cDNA encoding patchoulol synthase (*PTS*) was sequence optimized for expression in *S*. *cerevisiae* (synthesized by Geneart) and is based on the cDNA of *Pogostemon cablin* Benth. [GenBank: AY508730.1]. *PTS* was amplified using primers MF0018 and MF0019 adding overlaps homologous to plasmid pSP-GM1, while pSP-GM1 was linearized using restriction enzymes *Pac*I and *Not*I. Both were transformed into yeast, the plasmid pSP-P was isolated and analyzed for correct PCR product size and sequenced using primers MF0016_S and MF0017_S. The truncated version (first 1,590 bp were removed, start codon was reintroduced) of the endogenous yeast gene of HMG-CoA reductase (t*HMG1)* was PCR amplified using primers EG_tHMG_Fw and EG_tHMG_Rv and genomic DNA of yeast as template. The fragment was transformed into yeast together with pSP-P (linearized with *Xho*I) and pSP-Pt was isolated and verified using primers MF0014_S and MF_0015_S. The endogenous yeast gene of farnesyl diphosphate synthase (*ERG20*) was amplified from genomic yeast DNA using primers EMG_FPPS_Fw and EMG_FPPS_Rv. The fragment was transformed into yeast together with pSP-Pt (linearized with *Not*I) generating a fusion of farnesyl diphosphate synthase and patchoulol synthase via a short flexible linker (GSG) [18]. pSP-FPt was isolated and verified using primers EMG_FPPS_S and MF0017_S. The yeast strain *S*. *cerevisiae* CEN.PK2-1C was transformed with the constructed plasmids as described above.

ATP-citrate lyase of *Arabidopsis* sp. consists of two subunits. The cDNAs of both subunits, *ACLA*-1 and *ACLB-2*, were amplified from pYX042-ACLA-1 and pYX012-ACLB-2 [16] using primers EMG_ACL_3_PHXT7+ACLA-1 and EMG_ACL_4_ACLA-1+T2A for *ACLA-1* and primers EMG_ACL_5_T2A+ACLB-2 and EMG_ACL_6_ACLB-2+TTEF for *ACLB-2* which generates a separation of both cDNAs by a T2A sequence from *Thosea asigna* virus [19]. The truncated *HXT7* promoter (-392 bp to -1 bp) was amplified from p423H7 using primers EMG_ACL_1_pSP+PHXT7 and EMG_ACL_2_PHXT7+ACLA-1. Moreover, the *TEF* terminator was amplified from pUG6 using primers EMG_ACL_7_ACLB-2+TTEF and EMG_ACL_8_TTEF+AscI+pSP. The four fragments were transformed into yeast together with pSP-FPt (linearized with *Asc*I) for homologous recombination. The new plasmid pSP-FPt-ACL was isolated from yeast and verified using primers EMG_ACL_9_S and EMG_ACL_10_S, EMG_ACL_11_S and EMG_ACL_12_S, EMG_ACL_13_S and EMG_ACL_14_S.

### Protein extraction, SDS-PAGE and Western blot analysis

Cells corresponding to 100 mg CDW were resuspended in 1 mL 50 mM Tris, 1 mM MgCl_2_, 0.1 mM EDTA, 1 mM DTT, 1 mM PMSF, 1 μg/mL aprotinin, 1 μg/mL leupeptin, pH 7.2 and approx. 500 μL glass beads (0.75–1 mm diameter) were added before vortexing twice for 10 min. Samples were centrifuged (10 min, 16,000 *g*, 4°C) and the protein concentration of the supernatant was determined using a colorimetric assay after Bradford [20] using bovine serum albumin as reference. Sodium dodecyl sulfate polyacrylamide gel electrophoresis (SDS-PAGE) was performed according to Laemmli [21] using a 4% stacking gel and 12.5% separating gel. Samples according to 20 μg protein were loaded per slot on a gel together with a protein marker (Page Ruler^™^ Plus Prestained Protein Ladder, Thermo Scientific). Proteins were transferred to a nitrocellulose membrane at 0.8 mA/cm^2^ for 1 h. After blocking for 1 h with PBS buffer containing Tween 20 and milk powder, the membranes were incubated with the primary antibody (1:1000 dilution of Anti-2A Peptide Antibody, polyclonal from rabbit, Cat. #ABS31, Lot # 2446870, Merck Millipore; validated previously [19]) at 4°C overnight, washed and incubated with the secondary antibody (Anti-Rabbit IgG, Sigma Aldrich) for 1 h according to the manufacturer’s instructions. After washing, proteins were detected using a colorimetric assay with 5-bromo-4-chloro-3-indolyl phosphate and nitroblue tetrazolium (BCIP/NBT) in 50 mM sodium carbonate buffer.

### Yeast strain construction

The disruption of α-ketoglutarate dehydrogenase gene (*KGD1*) was performed using recyclable marker gene disruption cassettes as described by Güldener *et al*. [22]. The loxP flanked resistance cassette (loxP-P_*TEF1*_-kanMX-T_*TEF1*_-loxP) was amplified via PCR (Phusion^®^ High-Fidelity DNA Polymerase, Finnzymes/Thermo Scientific) from pUG6 using primers EMG_KGD_Fw and EMG_KGD_Rv (see S2 Table for primer sequences) generating approx. 50 bp overlap regions homologous to the genomic locus to be disrupted. Yeast was transformed with 2 μg PCR product and strains carrying the *kgd1* disruption were selected based on their resistance to G418. Moreover, genomic DNA was isolated and the correct integration was validated via PCR (*Taq* DNA Polymerase 1.1x Master Mix Red, Ampliqon, biomol) and sequencing using primers EMG_KGD_S1 and EMG_KGD_S2. The strain was transformed with pSH47 and Cre recombinase expression was induced in an overnight culture with 2% galactose for 30 min. Subsequently, cells were selected on agar plates with and without G418 as efficient recombination leads again to sensitivity to G418. The loss of the kanMX cassette was verified as well via PCR using primers EMG_KGD_S1 and EMG_KGD_S2. Finally, cells were streaked on SD agar plates containing 2 g/L 5-fluoroorotic acid (5-FOA) and 80 mg/L uracil to select for those strains that have lost the plasmid pSH47. The newly generated yeast strain carrying the α-ketoglutarate dehydrogenase gene disruption (*S*. *cerevisiae* CEN.PK2-1C: *MAT* **a**; *ura3-52 MAL2-8* ^*C*^ *SUC2 kgd1Δ*::*loxP*) was transformed with plasmids as described above.

### 2-phase cultivations

For characterizations of terpenoid production, yeast strains carrying plasmids were cultivated in SD medium. Cells were streaked from glycerol stocks onto agar plates, incubated for 3–4 days and used as inoculum for the first preculture (1/10 to 1/5 medium filling). The cells were harvested via centrifugation (5 min, 2,000 *g*, 4°C) after approx. 24 h, resuspended in 0.9% KCl and used to inoculate the second preculture to OD_660_ of 0.2–0.4 (1/5 medium filling). After incubation overnight, the second preculture was harvested and cells were resuspended in 0.9% KCl and used to inoculate the main cultures. For strain characterization during growth on glucose in batch mode as well as during growth on the produced side products ethanol, glycerol and acetate, main cultures (250 mL flasks with 1/5 medium filling) were inoculated to OD_660_ of 0.4 and 7.5% dodecane was added immediately to the cultures. As the fragrance compound patchoulol, the chosen sesquiterpenoid reporter, has volatile and potentially toxic properties, a two-phase cultivation was established as *in situ* product removal tool. Dodecane was chosen as it is widely used as second organic phase [19, 23, 24] due to its hydrophobicity (log P_O/W_ of 6.6 is regarded as biocompatible [25]), low volatility and good phase separation. Carbon source consumption was assessed to harvest the cultures when glucose was abolished (14–17 h) or ethanol, glycerol and acetate (4 d and 6 d). For glucose-limited experiments, a third preculture (250 mL flasks with 1/5 medium filling) was inoculated to OD_660_ of 0.4 and cultured for 15 h. Cells were harvested, washed in 0.9% KCl, resuspended in SD medium without glucose and used for the main culture (OD_660_ of 4.5, 500 mL flasks with 1/10 initial filling, 1/5 final filling). 7.5% dodecane was added immediately and carbon limiting conditions were realized by feeding glucose (SD medium containing 0.2% glucose x H_2_O) to the cultures at a rate of 51.5 μL/min for approx. 16 h using a multichannel peristaltic pump (IPC, ISM937, Ismatec). Cultivations were performed at least in triplicate.

### OD and cell dry weight determination

The optical density (OD) of cultivation samples was determined at 660 nm in duplicate by using a UV-1800 Shimadzu UV spectrometer. Cell dry weight (CDW) was determined by using an OD-CDW correlation: 0.4939 x OD_660_ = g/L CDW. For this purpose, the OD_660_ was determined in duplicate of 5 different samples of a cultivation and pellets of defined aliquots of each sample (triplicates) were dried at 75°C for 6 days and weighed.

### Metabolite and sugar analysis

Samples from the aqueous phase from cultivations were centrifuged (10 min, 16,000 *g*, 4°C) and the supernatant was frozen at -20°C if not measured directly. Glucose concentrations were estimated using Combur3Test^®^ (Roche) test stripes. Glucose and extracellular metabolites were quantified using HPLC/RID. An Agilent Technologies HPLC system, equipped with an RID detector at 35°C, was used. Compounds were separated on a Hi-Plex H column (300 x 7.7 mm, ID 6.5 mm, particle size 8 μm) with 5 mM sulfuric acid as isocratic mobile phase with a flow of 0.5 mL/min at 65°C for 38 minutes. The injection volume was 20 μL. Glucose, ethanol, glycerol and acetate were quantified using standard curves of the reference compounds.

### Analysis of sesquiterpenoids in the organic layer

Cultures were harvested by centrifugation (10 min at 5,000 *g*, 4°C); a sample of the dodecane layer was taken and dried with anhydrous Na_2_SO_4_, centrifuged for a second time and stored at -20°C if not measured directly. 100 μL of sample were diluted in 400 μL of n-hexane including 250 μM α-humulene (Sigma Aldrich) as internal standard and measured using GC/FID. An Agilent Technologies 7890A GC system equipped with a flame ionization detector (FID; 300°C, H_2_ flow set to 30 mL/min, air flow set to 400 mL/min, make-up gas (N_2_) flow set to 30 mL/min) was used for terpenoid quantification. A FactorFour VF-5ms column (CP8944; 30 m x 0.25 mm, ID 0.25 μm) was used and 1 μL sample was injected in splitless mode (inlet at 250°C). The carrier gas (H_2_) flow was set to 1.4 mL/min. The initial oven temperature was set to 80°C; after one min the oven temperature was increased to 120°C at a rate of 10°C/min. Thereafter, the temperature was increased to 160°C at a rate of 3°C/min and then to 180°C at a rate of 10°C/min. The oven temperature was finally increased to 270°C at a rate of 30°C/min and held for 2.5 min. Patchoulol was quantified using standard curves of a reference compound (patchouli alcohol, PhytoLab). Measurements were performed in duplicate. Additional sesquiterpenoids formed by the patchoulol synthase with nominal masses of 222 and 204 were identified by GC/MS and the comparative chemical composition of sesquiterpenoids was performed using FID using a 7890A GC system (Agilent Technologies). A HP-5MS column (30 m x 0.25 mm, ID 0.25 μm) was used and 1 μL sample was injected in splitless mode (inlet at 250°C). The carrier gas (He) flow was set to 1.1 mL/min. The oven temperature program described above was used. The ion source was kept at 230°C, the detection was performed in electron impact ionization mode at 70 eV and the mass spectrum was recorded from 50 to 550 m/z.

### Sterol extraction and analysis

A modified protocol after Asadollahi *et al*. [6] was used. Cells were harvested by centrifugation (4,000 *g*, 10 min) and the cell pellet was washed and then resuspended in dH_2_O. Cells corresponding to 30 mg CDW were pelleted again and resuspended in 4 mL 0.2 M HCl and incubated for 1 hour at 85°C in a water bath. Samples were cooled to room temperature and centrifuged (10 min, 4,000 *g*). The cell pellet was resuspended in 2 mL methanol and 1 mL 4 M KOH, transferred to a dark glass vials and incubated at 85°C for 2 hours in a water bath. Samples were cooled to room temperature, 5 mL heptane including 250 μM α-tocopherol (Sigma Aldrich) as internal standard was added and vortexed for 2 min. The heptane phase was taken for analytics. Ergosterol was quantified using HPLC/DAD and squalene was quantified using GC/FID, both via standard curves of the reference compounds (Sigma Aldrich). An Agilent Technologies HPLC system equipped with a 1260 Infinity DAD was used. Compounds were separated on a Poroshell 120 EC-C18 column (2.1 x 100 mm, 2.7 μM) with acetonitrile as isocratic mobile phase with a flow of 7 mL/min at 35°C for 6 minutes. The injection volume was 10 μL. Ergosterol and α-tocopherol were detected at 280 nm. The GC/FID system described in ‘Analysis of sesquiterpenoids in the organic layer’ was used with a modified oven temperature program. The initial oven temperature was set to 80°C; after one min the oven temperature was increased to 270°C at a rate of 10°C/min and held for 20 min. GC measurements were performed in duplicate.

### Fatty acid extraction, derivatization and analytics

A modified protocol after Chen *et al*. [26] was used. Cells corresponding to 5 mg CDW were resuspended in 500 μL 10 mM Tris pH 7.5, 10 μL 2-mercaptoethanol and 200 U lyticase (from *Arthrobacter luteus*, Sigma Aldrich) and incubated at 30°C for 30 min. Approx. 500 μL of glass beads (0.75–1 mm diameter), another 500 μL buffer and 200 μL acetic acid were added and vortexed two times for 5 min. 10 μL of 10 mg/mL heptadecanoic acid (Sigma Aldrich) dissolved in chloroform were added as internal standard. 3 mL of a chloroform-methanol (2:1) mixture was added, vortexed vigorously for 30 sec, centrifuged (10 min, 5,000 *g*, 4°C) and the generated chloroform layer was collected. Additional 3 mL of the chloroform-methanol mixture were added to the aqueous and cell debris layer, vortexed and centrifuged again. The chloroform layer were combined and evaporated to dryness using a rotary evaporator (Rotavapor^®^ R-210, Büchi) and afterwards a desiccator with silica gel orange overnight. The dried lipid residue was dissolved in 500 μL 10% BF_3_-methanol (Sigma Aldrich) and incubated in sealed screw cab tube at 90°C for 20 min. After cooling down to room temperature, 300 μL of a saturated NaCl solution was added and vortexed. Fatty acid methyl esters were extracted with 600 μL n-hexane via harsh vortexing and analyzed using GC/FID. The GC/FID system described in ‘Analysis of sesquiterpenoids in the organic layer’ was used with a modified oven temperature program. The initial oven temperature was set to 50°C; after one min the oven temperature was increased to 270°C at a rate of 7°C/min and held for 10 min. Fatty acid methyl esters (F.A.M.E.) mix C_8_ –C_24_ (CRM18918 Supelco) as well as GC-MS (system described in ‘Analysis of sesquiterpenoids in the organic layer’, oven temperature program identical to the one from GC/FID) were used for identification of fatty acid methyl esters.

## Results

### Sesquiterpenoid production in *S*. *cerevisiae*: evaluation of two-phase cultivation conditions and sesquiterpenoid spectrum

**Fig 2:**
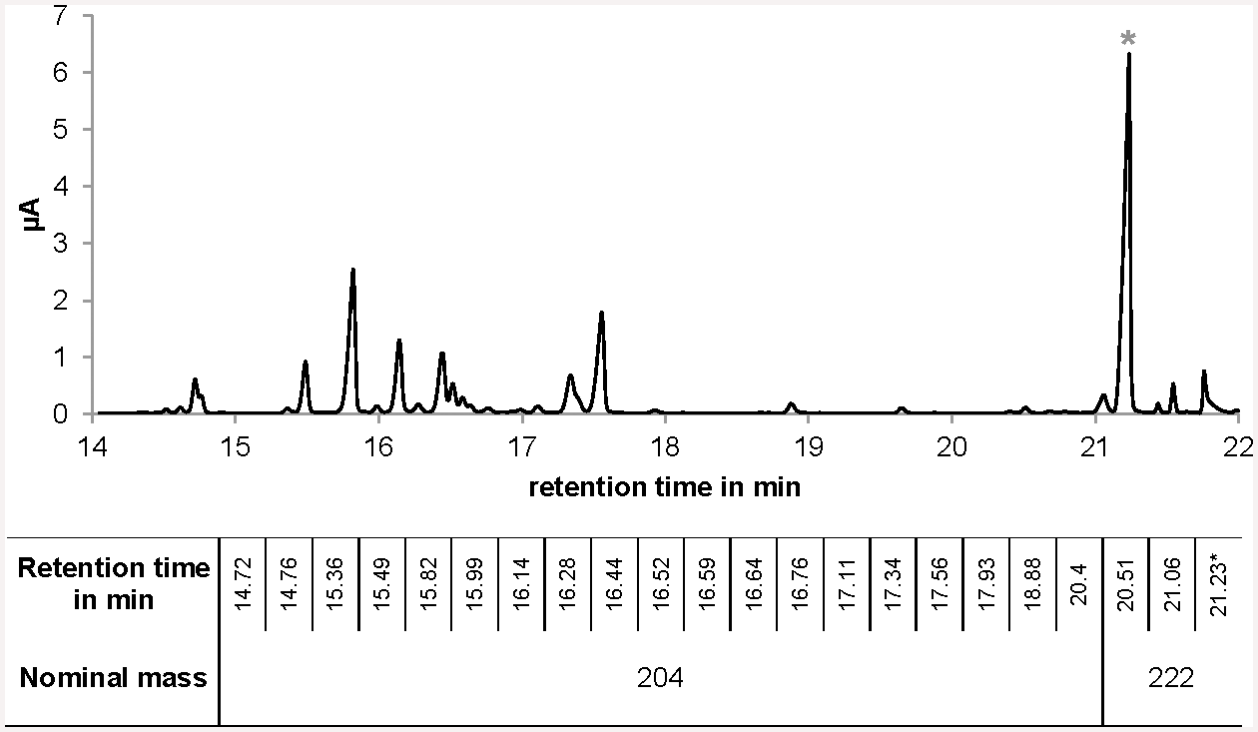
Sesquiterpenoid spectrum of patchoulol synthase produced by yeast. A representative GC/FID analysis of the patchoulol synthase products in the organic phase of a yeast two-phase cultivation heterologously expressing patchoulol synthase is shown. 22 Peaks with nominal masses of 222 (sesquiterpenoids with hydroxy-group) and 204 (sesquiterpenoids without hydroxy-group) were detected. The peak marked with an asterisk (*) was identified as patchoulol.

Since the fragrance compound patchoulol, the chosen reporter sesquiterpenoid, has volatile and potentially toxic properties, a two-phase cultivation using dodecane was established. Dodecane showed no detrimental effects on physiological parameters (cell growth, glucose consumption, ethanol, glycerol and acetate formation) or ergosterol production when 7.5% were added to yeast cultures carrying pSP-GM1 as empty vector (S1 Fig). Moreover, dodecane captures patchoulol efficiently over time and prevents loss in the air (S2 Fig). Thus, a two-phase cultivation using dodecane is well suited. *S*. *cerevisiae* was transformed with plasmid pSP-P carrying a synthetic gene coding for patchoulol synthase under control of *TEF1* promoter and was analyzed for patchoulol production using the two-phase cultivation procedure with dodecane. Patchoulol was produced and its accumulation in the organic phase was monitored over time. Patchoulol formation curve and biomass formation curve run in parallel and both stagnate as soon as glucose is consumed (S3 Fig). Moreover, additional sesquiterpenoids were detected. Using a long-time experiment, high patchoulol concentrations were achieved and within these samples 22 peaks with nominal masses of 222 (sesquiterpenoids with hydroxy-group) and 204 (sesquiterpenoids without hydroxy-group) could be detected (Fig 2). Based on a comparative chemical composition of sesquiterpenoids produced, patchoulol represents 34.3% +/- 1.6 of total sesquiterpenoids. The patchoulol content determined here is used hereafter to estimate the production of total sesquiterpenoids in this work.

### Engineering of terpenoid pathway: t*HMG1* overexpression and fusion of FPP synthase with patchoulol synthase

Deregulating the MVA pathway is a prerequisite for metabolic engineering approaches to redirect the carbon flux within the central carbon metabolism towards terpenoids. The HMG-CoA reductase (Hmg1/2p) is described as the most important site of regulation in the MVA pathway. The overexpression of a truncated, and thus soluble, version (t*HMG1*) that is devoid of feedback inhibition by FPP (farnesyl diphosphate) has been shown to enhance terpenoid production several times [5, 7, 27–29]. Thus, t*HMG1* was overexpressed using pSP-Pt carrying t*HMG1* under control of *PGK1* promoter next to *PTS* under control of *TEF1* promoter.

**Fig 3:**
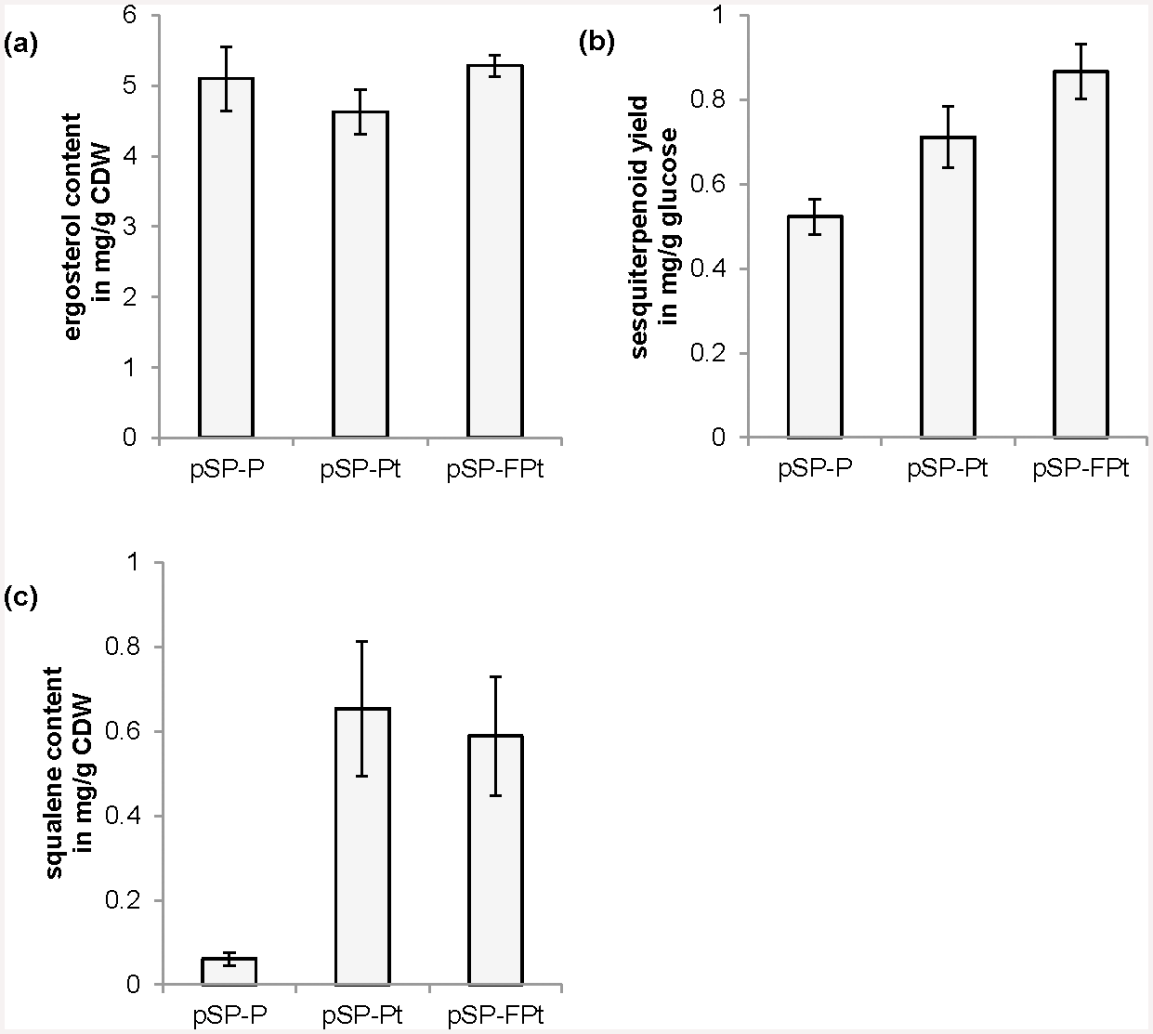
Effect of t*HMG1* overexpression and fusion of FPP synthase and patchoulol synthase on terpenoid production. Terpenoid formation of yeast strains carrying pSP-P, pSP-Pt and pSP-FPt after growth on glucose in batch conditions: (a) ergosterol content, (b) sesquiterpenoid yield on glucose and (c) squalene content. Shown are mean values and standard deviations of six experiments.

The generated yeast strain showed a slightly reduced growth rate accompanied by a slightly reduced glucose consumption rate as well as ethanol, glycerol and acetate formation rate (S4 Fig). The ergosterol content was basically unchanged but the sesquiterpenoid yield on glucose was indeed increased by 36% (see Fig 3a and 3b). Nevertheless, the strongest effect was visible for squalene. The squalene content was increased about 10-fold (see Fig 3c).

Patchoulol synthase competes with squalene synthase (Erg9p) for their common substrate FPP. However, Erg9p has a lower K_m_ and a significantly higher k_cat_ than Pts (K_m_ of Erg9p for FPP is 2.5 μM while K_m_ of Pts for FPP is 4.5–6.8 μM; k_cat_ of Erg9p is 0.53 s^-1^ while k_cat_ of Pts is 0.00043–0.026 s^-1^ [30–32]). The physical fusion of FPP synthase and Pts is thought to increase the flux towards sesquiterpenoids due to close proximity of the active sites of the enzymes which could bypass loss of the intermediate FPP by diffusion, degradation or conversion through competitive pathways via substrate channeling [33]. Thus, a fusion construct was generated according to Albertsen *et al*. [18] and overexpressed under the control of *TEF1* promoter using pSP-FPt which carries additionally t*HMG1* under control of *PGK1* promoter. The generated strain exhibits a further decreased growth and glucose consumption rate as well as metabolite formation rate (S4 Fig). The use of the fusion protein had basically no effect on squalene and ergosterol levels but it led to an increase in sesquiterpenoid yield on glucose by 22% (see Fig 3).

### Choice of carbon source: impact on terpenoid production

The focus of this study was on terpenoid production based on glucose. However, yeast is producing ethanol, glycerol and acetate while consuming and growing on glucose in batch mode (hereafter: glucose-phase). These non-fermentable carbon sources can then be consumed and used as carbon source by yeast after the diauxic shift in a second growth phase (hereafter: ethanol-phase). Thus, terpenoid production was not only analyzed during growth on glucose but as well during growth on those non-fermentable carbon sources.

**Fig 4:**
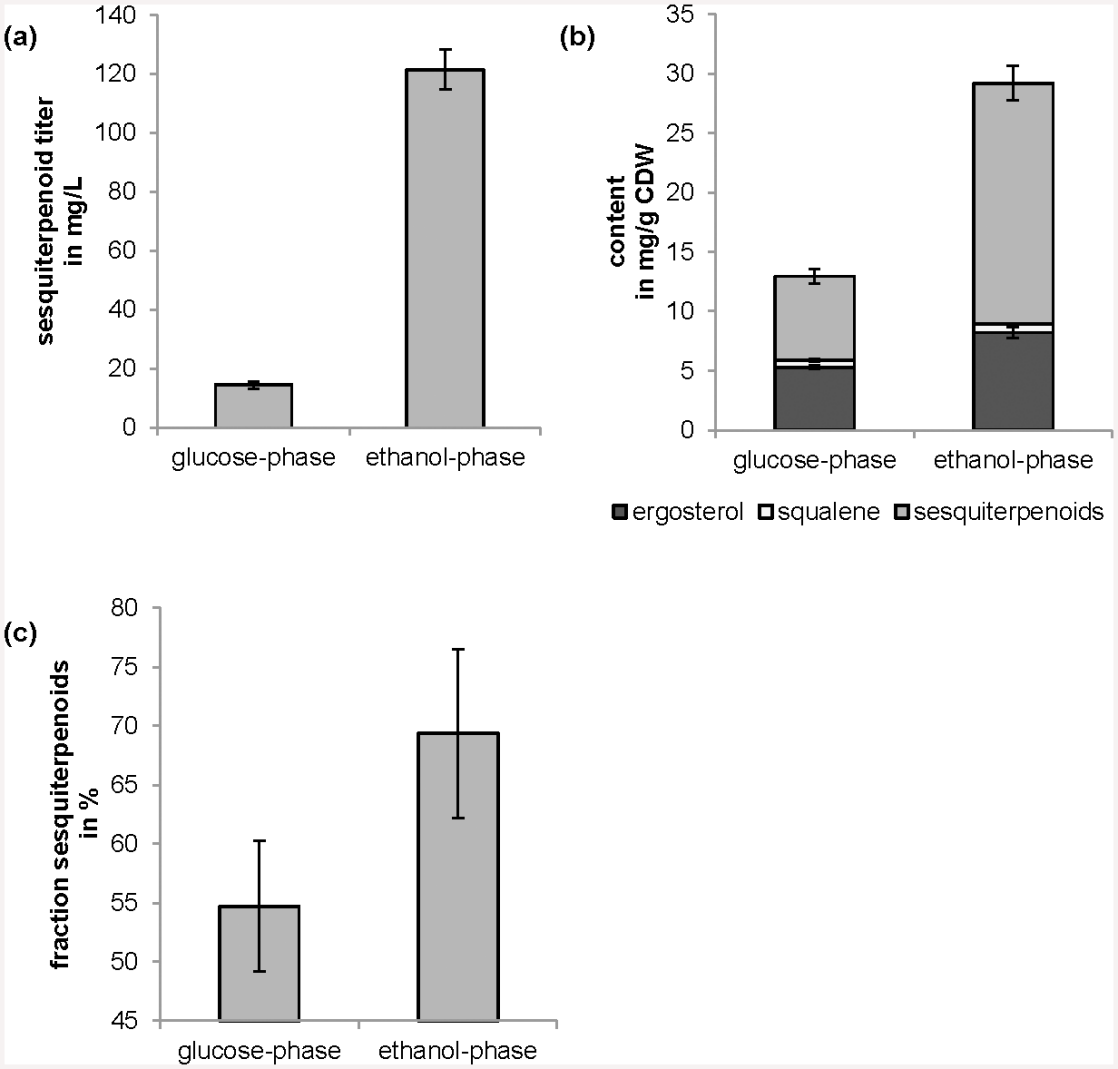
Effect of carbon source on terpenoid production. Terpenoid production of yeast strain carrying pSP-FPt during glucose-phase (cells were harvested as soon as glucose was exhausted) and during ethanol-phase (cells were harvested after four days when ethanol, glycerol and acetate were exhausted). (a) sesquiterpenoid titer; (b) ergosterol, squalene and sesquiterpenoid content per cell dry weight, CDW; (c) fraction of sesquiterpenoids in total terpenoids (ergosterol, squalene and sesquiterpenoids). Shown are mean values and standard deviations of three experiments.

The yeast strain expressing the fusion protein of FPP synthase and patchoulol synthase was cultured on glucose in batch mode. As expected, cells grew first on glucose while producing ethanol, glycerol and acetate and then cells shifted to growth on the fermentation metabolites and the carbon sources were consumed completely. Interestingly, sesquiterpenoid titer was increased tremendously by a factor of 8.4 from the glucose- to the ethanol-phase (Fig 4a). To account for different biomass formation, the sesquiterpenoid yield was calculated per biomass (mg sesquiterpenoid/g CDW, cell dry weight). The yield was increased by a factor 2.9 in the ethanol-phase (Fig 4b). The ergosterol and squalene contents were also increased during ethanol-phase by a factor 1.6 and 1.2 respectively. Furthermore, the fraction of sesquiterpenoids in total terpenoids (sesquiterpenoids plus squalene plus ergosterol) was increased from 55% in glucose-phase to even 69% in ethanol-phase (Fig 4c).

### Metabolic engineering to redirect carbon flux towards terpenoids: disruption of *KGD1* of citric acid cycle

Based on our *in silico* computation [8], a gene knockout within the citric acid cycle in combination with avoidance of ethanol and acetate production is a promising metabolic engineering target. The α-ketoglutarate dehydrogenase gene (*KGD1*) of α-ketoglutarate dehydrogenase complex was chosen as knockout target. The gene was disrupted in *S*. *cerevisiae* using the Cre-LoxP system via integration of a resistance cassette at the genomic locus and thus replacement of the first 1,702 bp of the gene. The resistance cassette was cut out using Cre recombinase leaving a single LoxP site at the genomic locus. The strain carrying the *KGD1* disruption was transformed with pSP-FPt for characterization in the two-phase cultivation.

**Fig 5:**
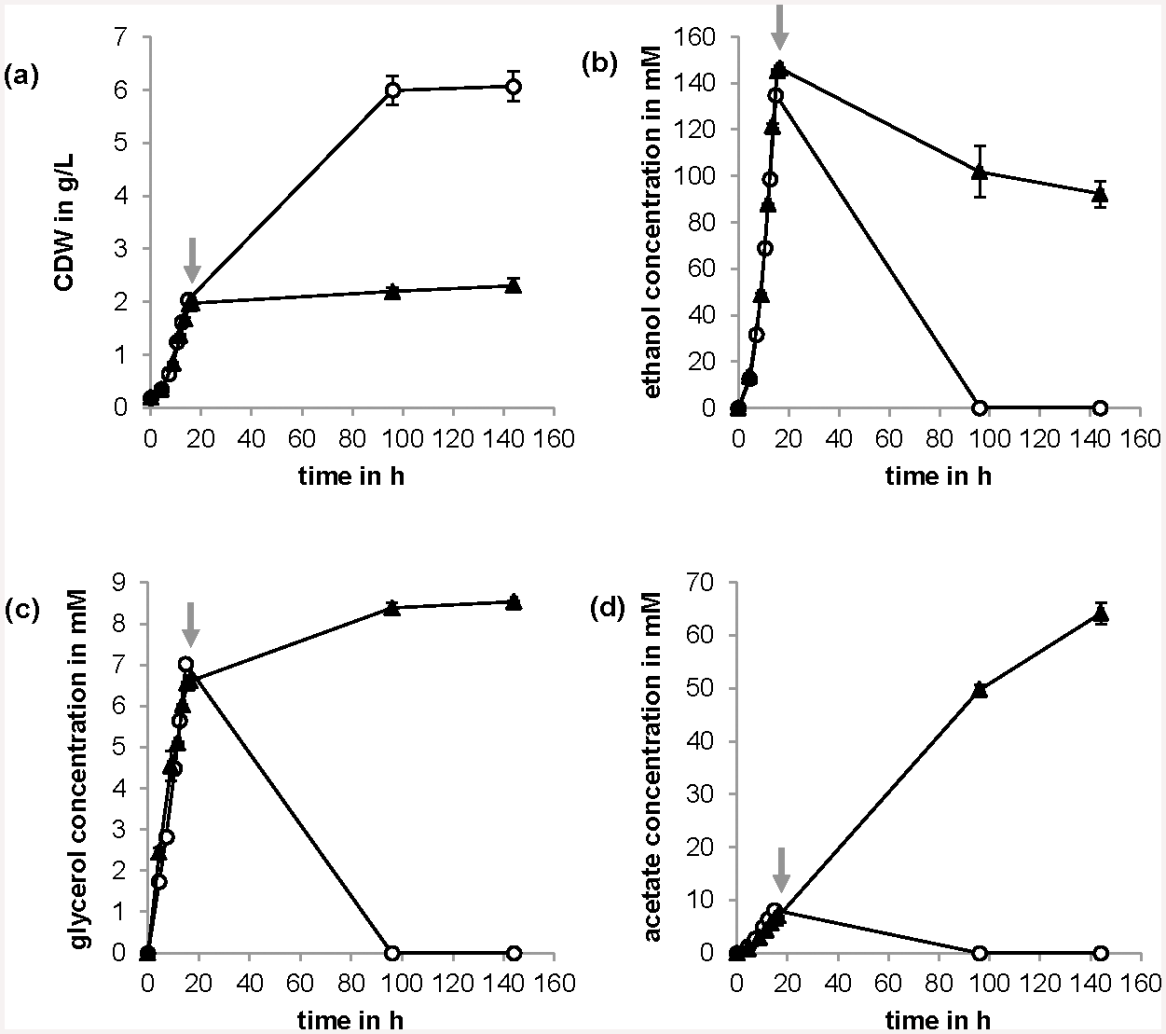
Effect of disruption of *KGD1* on physiological parameters of yeast. Influence of *KGD1* disruption (▲, *kgd1*Δ) in comparison to the wild type (○) on: (a) biomass formation (cell dry weight, CDW), (b) ethanol formation, (c) glycerol formation and (d) acetate formation as a function of time during glucose-phase as well as ethanol-phase, arrows indicate the point in time when glucose was exhausted. Shown are mean values and standard deviations of three experiments.

**Fig 6:**
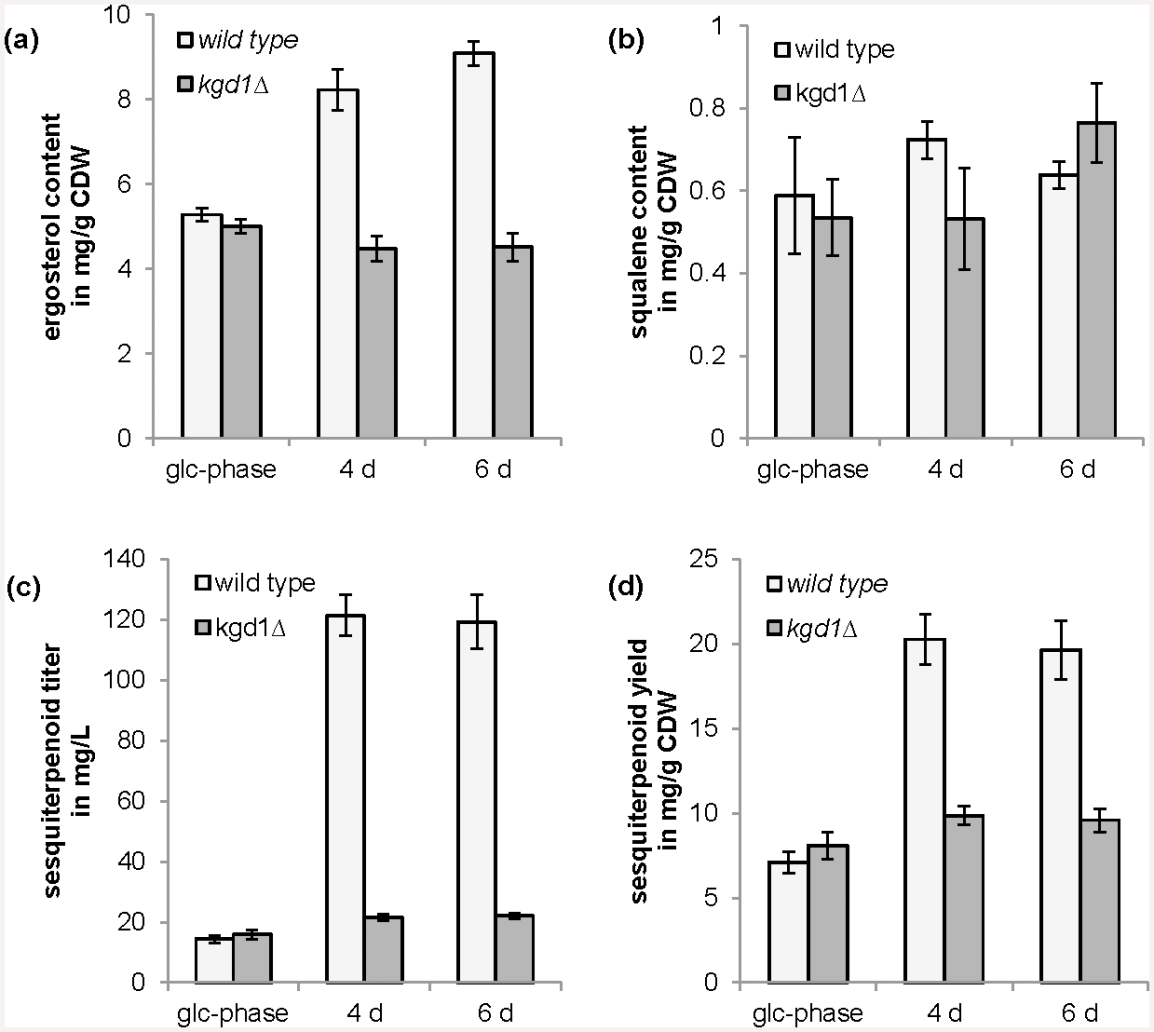
Effect of disruption of *KGD1* on terpenoid production. Influence of *KGD1* disruption in comparison to the wild type on: (a) ergosterol content, (b) squalene content, (c) sesquiterpenoid titer and (d) sesquiterpenoid yield on biomass during glucose-phase (glc-phase) and ethanol-phase at four and six days (4 d, 6 d). Shown are mean values and standard deviations of three experiments.

Disruption of *KGD1* did not have any significant effect on physiological parameters (growth rates, or ethanol, glycerol and acetate formation), ergosterol and squalene content or sesquiterpenoid production during growth on glucose in batch mode until glucose was consumed (see Figs 5 and 6). However, cell growth was interrupted after glucose was consumed while the wild type strain grew further on ethanol, glycerol and acetate as carbon sources. After 4 days, the wild type strain had consumed ethanol, glycerol and acetate completely. The strain carrying the *KGD1* disruption, however, consumed ethanol only partially while producing glycerol and especially high amounts of acetate (Fig 5d). This trend continued after 6 days, where some ethanol was consumed, while acetate was produced in high amounts. Acetate concentrations were increased nearly 10-fold from glucose-phase to ethanol-phase after 6 days with this mutant. Squalene content was not significantly affected while ergosterol content was decreased in the strain carrying the *KGD1* disruption in the ethanol-phase (Fig 6a and 6b). Surprisingly, sesquiterpenoid titer or yield per CDW was reduced in this strain (Fig 6c and 6d).

### Heterologous pathway to synthesize cytosolic acetyl-CoA: ATP-citrate lyase

The pyruvate dehydrogenase bypass, especially acetate activation, appears to be a central issue *in vivo* in supplying significant acetyl-CoA for terpenoid production. The enzyme ATP-citrate lyase was identified as promising metabolic engineering target for increased terpenoid production due to increased energy-efficiency in comparison to the pyruvate dehydrogenase bypass [8]. Moreover, the heterologous expression of this enzyme could circumvent acetate accumulation.

**Fig 7:**
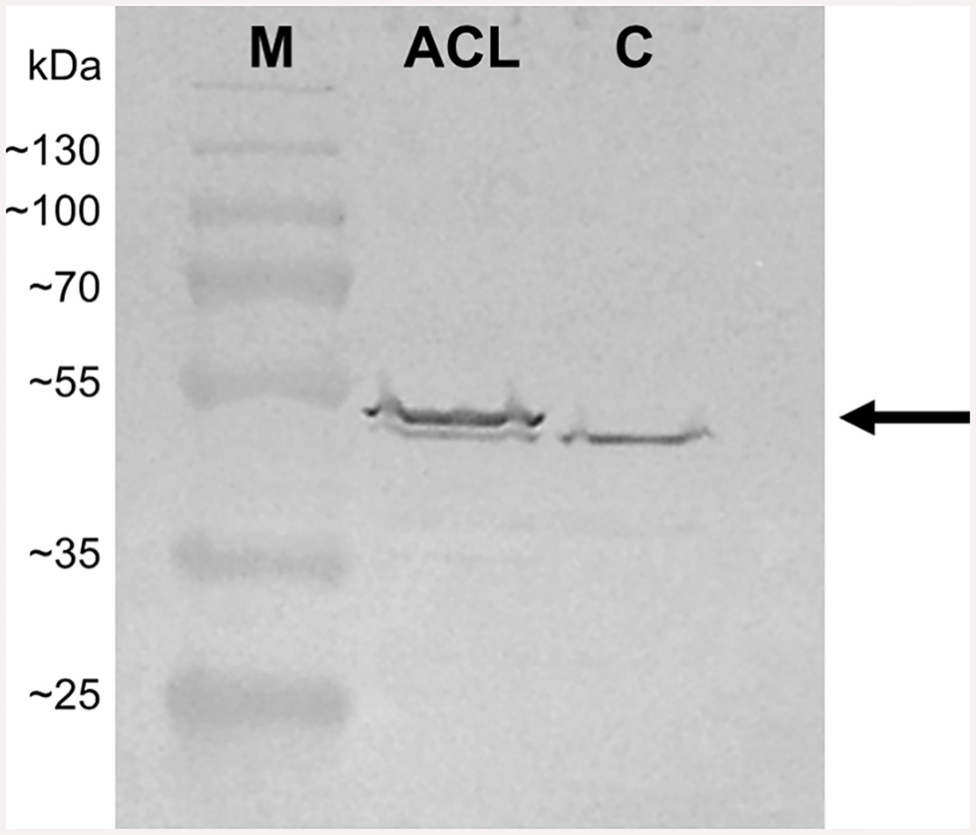
Western blot analysis of yeast proteins. A representative western blot of protein from yeast strain carrying pSP-FPt-ACL (ACL) and pSP-FPt as control (C) is shown. The protein ladder is shown on the left (M). The predicted position of AclA-1-T2A is indicated with an arrow.

Only few data on kinetic properties of ATP-citrate lyase from different organisms are available. Even though no data are available for ATP-citrate lyase from Arabidopsis, this enzyme has been shown to be active in *S*. *cerevisiae* [16], and was therefore chosen for expression in this study. The enzyme is made up of two different polypeptide chains and consequently two genes; the cDNAs of *ACLA-1* and *ACLB-2* were used within this study. To reduce cloning time and effort and avoid excessive promoter and terminator use, the cDNAs were separated by a T2A sequence from *Thosea asigna* virus for polycistronic expression and introduced to pSP-FPt generating pSP-FPt-ACL. The *in vivo* functionality of the T2A sequence to generate two separate proteins was assessed via western blotting using 2A-peptide specific antibodies. Fig 7 shows a western blot of cell lysates of *S*. *cerevisiae* carrying pSP-FPt-ACL and pSP-FPt as a control. The blot should reveal a band corresponding to a molecular weight of 51 kDa if the polypeptide was cleaved correctly to AclA-1-T2A and AclB-2. A band corresponding to a slightly lower molecular weight was visible in protein from both the strain expressing the polycistronic ATP-citrate lyase construct and the control strain not expressing the enzyme. Yet western blotting indeed revealed an additional band at the correct height only for the strain expressing the polycistronic ATP-citrate lyase construct.

**Fig 8:**
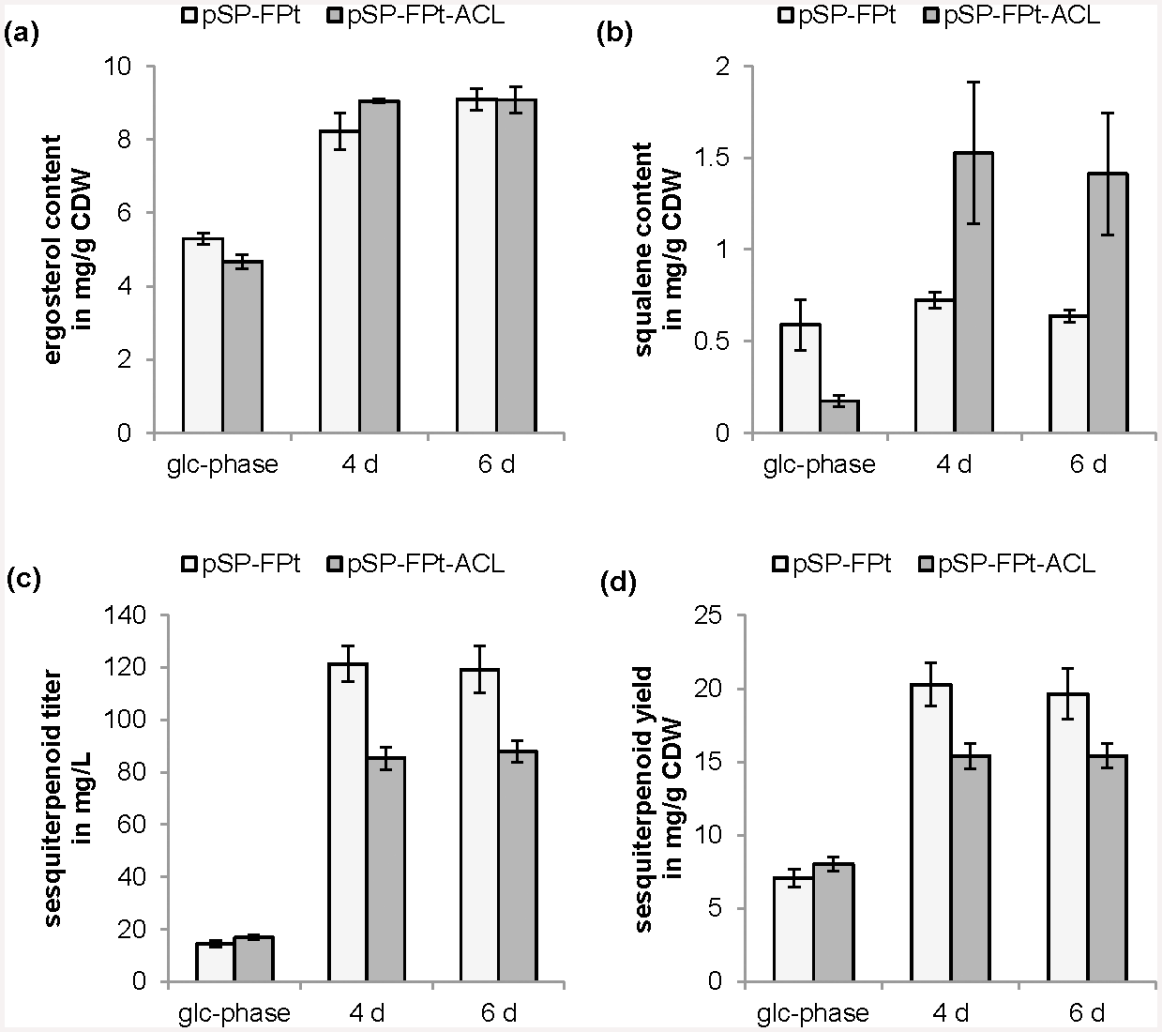
Effect of ATP-citrate lyase (*ACL*) overexpression on terpenoid production. Terpenoid formation, *i*.*e*. (a) ergosterol content, (b) squalene content, (c) sesquiterpenoid titer and (d) sesquiterpenoid yield on biomass of yeast strains carrying pSP-FPt and pSP-FPt-ACL during glucose-phase (glc-phase) and ethanol-phase at four and six days (4 d, 6 d). Shown are mean values and standard deviations of three experiments.

The *S*. *cerevisiae* strain expressing the polycistronic ATP-citrate lyase construct which generates these two separate polypeptides was characterized in the two-phase cultivation conditions. ATP-citrate lyase overexpression did not have any effect on physiological parameters (growth, glucose consumption, ethanol, glycerol, and acetate formation) at the cultivation conditions investigated: batch conditions in glucose- and ethanol-phase (S5 Fig). The effect on terpenoid production was ambivalent. Only a minor effect could be detected on ergosterol content (Fig 8a), while squalene content was decreased in glucose-phase but increased in ethanol-phase (Fig 8b). Sesquiterpenoid production was basically not affected in glucose-phase, however, was even reduced during ethanol-phase (Fig 8c and 8d).

To evaluate whether the reduced sesquiterpenoid production during ethanol-phase could be explained by a redirection of the carbon flux from acetyl-CoA towards fatty acids instead of terpenoids, fatty acid content of cells was determined. However, no increase in fatty acid content of the yeast strain expressing the polycistronic ATP-citrate lyase construct could be detected (S6 Fig). To analyze whether activity and affinity of ATP-citrate lyase to its substrate are the limiting factors, citrate supplementation was tested. Supplementation of citrate to the medium (2.5 and 5 mM) to cells carrying pSP-FPt and pSP-FPt-ACL, caused a strong decrease in sesquiterpenoid production in glucose-phase (hardly detectable amounts) as well as in ethanol-phase (reduction to about half) while physiological parameters were not affected (S7 Fig).

## Discussion

Enhancing the intracellular capacity for heterologous production of acetyl-CoA derived products is not limited to terpenoids; metabolic engineering strategies could as well be applied to other acetyl-CoA derived products such as lipids, polyketides, polyhydroxyalkanoates or *n*-butanol. Currently, there is still room for improvement via metabolic engineering to boost product yield for all classes of these products. Recent studies have focused on modifications within the central carbon metabolism of *S*. *cerevisiae* to redirect the metabolic flux towards an increased cytosolic acetyl-CoA production, the precursor of the MVA pathway. Strategies employed in these studies include: engineering of the pyruvate dehydrogenase bypass (*ALD6* and a mutant *acs* from *Salmonella*) [34]; modification of ammonium assimilation (*gdh1Δ* and *GDH2*) [35, 36]; combining engineering of the pyruvate dehydrogenase bypass (*ALD6*, a mutant *acs* from *Salmonella* and *ADH2*) with deletions in the glyoxylate cycle (*cit2Δ* and *mls1Δ*) for terpenoid, biobutanol, polyhydroxybutyrate, 3-hydroxypropionic acid and fatty acid production [37–41]; expression of the phosphoketolase pathway for fatty acid and polyhydroxybutyrate production [41–43]; functional expression of a bacterial pyruvate dehydrogenase complex in the cytosol [44]; functional expression of the acetylating acetaldehyde dehydrogenase and pyruvate-formate lyase [45]; and a model for an indirect acetyl-CoA transport from mitochondria to the cytosol, including acetate:succinyl-CoA transferase (*Ach1*) was proposed [46]. Several strategies to improve terpenoid production were also predicted *in silico* in our previous study [8]. Here, we report the *in vivo* validation of selected strategies and identify general considerations important for the *in vivo* validation of such stoichiometric metabolic network predictions.

### Sesquiterpenoid spectrum

A two-phase cultivation procedure was established for the volatile sesquiterpenoid patchoulol. 21 additional sesquiterpenoids in addition to patchoulol could be detected as product of the patchoulol synthase. Here, patchoulol represents about 34.3% of total sesquiterpenoids produced by this enzyme, slightly lower than the previously reported value of 36.9% [32]. This difference is likely because we could detect more sesquiterpenoids due to a higher production titer. The value was used to estimate the production of total sesquiterpenoids using patchoulol as a marker. As prerequisites for validating metabolic engineering strategies within the central carbon metabolism, the flux from acetyl-CoA towards the desired product was increased and carbon sources were analyzed for their capacity to support sesquiterpenoid production.

### t*HMG1* overexpression and fusion of FPP synthase with patchoulol synthase have a small effect on sesquiterpenoid production

Two well described engineering strategies were employed to increase the flux from the central carbon precursor acetyl-CoA to the terpenoid product. A truncated HMG-CoA reductase (t*HMG1*) that is devoid of feedback inhibition by FPP was overexpressed to deregulate the MVA pathway. In addition, endogenous FPP synthase and patchoulol synthase were fused to increase the flux towards the desired product and to bypass loss of the intermediate FPP via substrate channeling. Surprisingly, overexpression of t*HMG1* led only to an increase in sesquiterpenoid yield by 36% and protein fusion led only to a further increase by 22%. However, squalene levels were increased approximately 10-fold when t*HMG1* was overexpressed. HMG-CoA reductase was identified as the rate-limiting enzyme in early sterol biosynthesis in yeast in the late 1990s when its overexpression was shown to increase squalene levels by 10-fold and 40-fold [27, 28]. However, these apparently strong improvements in squalene correspond to very low absolute amounts when compared to ergosterol and sesquiterpenoid amounts (Fig 4b). For most mono-, di-, and sesquiterpenoids the effect was comparably low with an increase of 1.4 to 5-fold [4, 5, 7, 36, 47–50] and in some cases, no effect or even an adverse effect was detected [4, 18, 48] depending on terpenoid synthase and previous genetic modifications. Several fusion proteins consisting of terpenoid synthase and FPP or GPP (geranyl diphosphate) synthase were shown to increase terpenoid production [33, 51, 52]. Albertsen and co-workers [18] showed that a fusion protein consisting of patchoulol synthase and endogenous FPP synthase can increase patchoulol production up to 2-fold, again depending on additional genetic modifications. Thus, our findings that t*HMG1* overexpression and fusion of FPP synthase with patchoulol synthase have small positive effects on sesquiterpenoid production are in good agreement with literature. Therefore, further production improvements observed from these modifications likely depend strongly on additional genetic modifications of the yeast strain used or even on cultivation conditions.

### Carbon source strongly influences terpenoid production

Yeast shows two separate growth phases in batch mode: a phase of growth based on glucose as carbon source accompanied by the production of ethanol, glycerol and acetate (glucose-phase), and a second growth phase based on ethanol, glycerol and acetate as carbon source (ethanol-phase). Sesquiterpenoid as well as total terpenoid (sesquiterpenoids plus squalene plus ergosterol) titer and yield on biomass were increased tremendously during growth in ethanol-phase in comparison to glucose-phase (Fig 4a and 4b). While Chen and co-workers [37] showed that at least 50% of sesquiterpenoids were produced during the glucose-phase in comparison to the following ethanol-phase, 88% of sesquiterpenoids formed after four days were produced during the ethanol-phase in our experiments (see Fig 4a). A patchoulol titer of 41.6 +/- 1.3 mg/L was achieved, which is to our knowledge the highest final titer of patchoulol in shake flasks and which is as high as the highest reported patchoulol titer in a bioreactor (40.9 +/- 2.7 mg/L) [18]. The higher potential of ethanol as a carbon source to increase terpenoid yield was indeed predicted in our *in silico* analysis [8] and demonstrated previously with ethanol feeding [53]. The results presented here indicate that the use of glucose as a cheap carbon source together with ethanol, which was produced by the yeast itself, is a promising approach to produce terpenoids in high yields.

Moreover, we could show that the fraction of sesquiterpenoids in total terpenoids was increased from 55% in glucose-phase to 69% in ethanol-phase after four days (Fig 4c). This increase indicates that not only the flux into the mevalonate pathway was increased during growth on ethanol, glycerol, and acetate but the partition of the flux at the intersection of FPP was affected positively, which has to our knowledge not been shown before.

### *In vivo* validation and considerations of *in silico* predicted strategies

The disruption of α-ketoglutarate dehydrogenase gene (*KGD1*) of α-ketoglutarate dehydrogenase complex did not lead to the desired increase in terpenoid production. The citric acid cycle exhibits only a weak activity during growth on high glucose concentrations [54] and the flux is redirected mainly towards ethanol (Crabtree effect) due to a complex regulatory machinery involving repression of *LPD1* of pyruvate dehydrogenase complex [55], aldehyde dehydrogenases *ALD2-4* [56], acetyl-CoA synthetase *ACS1* [57] and alcohol dehydrogenase *ADH2*, which converts ethanol to acetaldehyde, [58] at high glucose concentrations while pyruvate decarboxylase *PDC1* is presumed to be activated by glucose (or repressed by ethanol) [59, 60]. Hence the disruption of *KGD1* resulted in no effect. Our previous *in silico* prediction did not take this regulation into account [8]. However, during ethanol-phase, the use of a different carbon source circumvents this regulation and leads to a higher flux into the citric acid cycle. Here, disruption of α-ketoglutarate dehydrogenase gene under these conditions did lead to an effect (Fig 5). Cell growth was inhibited, high amounts of acetate were secreted, and terpenoid formation was decreased. In addition, glucose-limited conditions were analyzed to determine the effects of this modification during conditions, which normally increases the flux into the citric acid cycle [54]. Similarly to ethanol-phase cultures, the strain carrying the *KGD1* disruption exhibited reduced cell growth and production of acetate during glucose limitation, while the wild type strain did not produce any fermentation products. However, terpenoid formation was not affected under these conditions (S8 Fig).

Disruption of *KGD1* was predicted to redirect the metabolic flux from citric acid cycle via pyruvate dehydrogenase bypass towards terpenoids. Although the metabolic flux was indeed redirected to the bypass, the redirection was interrupted at the level of acetate (see Fig 1). Activation of acetate and acetyl-CoA formation seems to be the rate-limiting step in this process due to regulation and activity of acetyl-CoA synthetase (*ACS1*, *ACS2*). Shiba *et al*. [34] reported the same issue, and succeeded in reducing acetate formation while at the same time increasing terpenoid formation by overexpressing a non-post-translationally regulated acetyl-CoA synthetase from *Salmonella* together with a native aldehyde dehydrogenase. However, acetate formation could not be completely abolished with this strategy and acetate levels were more than three times higher in our experiments. Lian *et al*. [61] also reported similar issues of increased acetate formation to cytotoxic levels, which was accompanied by decreased product formation (*n*-butanol).

The disruption of α-ketoglutarate dehydrogenase led to no effect during batch conditions and to three consistent effects during glucose-limited conditions or ethanol-phase: (i) reduced growth during glucose-limited conditions or abolished growth during ethanol-phase, (ii) acetate formation, which was especially high in the ethanol-phase, and (iii) reduced terpenoid formation in ethanol-phase only. Strains carrying the gene disruption should theoretically be able to grow on glucose as well as on ethanol according to our *in silico* computations. However, a reduced growth of citric acid cycle knockouts on non-fermentable carbon sources has been described in literature [62]. An explanation for the effect has not been given in previous literature. Acetate formation could be the reason for the growth inhibition as acetate acts as a weak acid and dissipates the pH gradient over the plasma membrane, which in turn has to be balanced by ATP-consuming proton pumps [63]. This additional ATP requirement could as well be a reason for the reduced terpenoid production during the ethanol-phase, as terpenoid synthesis requires high amounts of ATP. Moreover, acetic acid has been described to trigger apoptosis (programmed cell death) in yeast even at pH levels above neutral. Apoptosis is likely triggered via reactive oxygen species, especially increased H_2_O_2_ levels, modulated by active superoxide (O_2_^−^) dismutase (which converts 2 O_2_^−^ + 2 H^+^ into H_2_O_2_ and O_2_) and via acetic acid inactivated catalase (which normally converts 2 H_2_O_2_ into H_2_O and O_2_). Additionally, increased proteolytic activity has been shown upon acetic acid stress [64, 65]. Acetate formation could also be the reason for the negative effect observed on terpenoid formation due to the role of acetate as a signal, which triggers multiple effects. Another weak organic acid is citrate. Citrate supplementation in our experiments led also to a strong reduction in terpenoid formation. Thus, there could be a common mechanism. Nevertheless, a yet unknown mechanism could add to the effects seen. Acetate was produced during glucose-limited conditions, however, concentrations were comparable to those in batch conditions. Thus, one can assume that an additional mechanism is responsible for the inhibition of growth. Further studies need to address the issue of reduced growth, reduced terpenoid formation and the effects of weak organic acids such as acetate to elucidate this issue.

It can be proposed from these findings that acetate formation should be avoided in order to increase terpenoid production. An alternative solution could be the use of heterologous pathways, which circumvent the pyruvate dehydrogenase bypass. ATP-citrate lyase converts citrate that has been produced via pyruvate dehydrogenase complex and the citric acid cycle in the mitochondria to acetyl-CoA. The reaction is more energy-efficient and avoids the pyruvate dehydrogenase bypass. ATP-citrate lyase from Arabidopsis was overexpressed as a polycistronic construct in this study. A self-cleaving T2A sequence from *Thosea asigna* virus was used for the generation of two separate polypeptides from a polycistronic mRNA. This represents only the second time such a strategy has been used in yeast biotechnology [19], and validates the efficiency of self-cleaving peptides to reduce cloning time and effort for expression of multiple transgenes from a single promoter.

Overexpression of ATP-citrate lyase from *Arabidopsis* did not lead to the desired circumvention of acetate formation or an increase in terpenoid production during batch cultivation conditions in either glucose- or ethanol-phase or even glucose-limited conditions (S9 Fig). In the ethanol-phase, squalene levels were indeed increased, however, sesquiterpenoid formation was decreased. Unfortunately, those decreased levels could not be explained by modified fatty acid formation. A negative influence of the enzyme tag from the viral sequence on activity seems improbable as ATP-citrate lyase genes from animals arose from gene fusion of the two genes (as found in *Arabidopsis sp*.) and thus possess a natural linker of the size of the tag used between the two polypeptide chains homologous to the plant ones [16].

ATP-citrate lyase has been overexpressed in yeast before to increase *n*-butanol or fatty acid production. The enzyme from mouse led to an increase in fatty acid formation of 15–20% [66]. The enzyme from *Arabidopsis* did not have an effect while the enzyme from *Yarrowia lipolytica*, an oleaginous yeast, led to a 2-fold increase in *n*-butanol production [61]. While no kinetic properties of ATP-citrate lyase from those organisms are available, we assume that the kinetic properties are unfavorable and *in vivo* activities are not sufficient of the enzyme from *Arabidopsis* or mouse to influence formation of acetyl-CoA derived products tremendously. Previously, Lian and coworkers [61] assumed that the *in vivo* activity and affinity of ATP-citrate lyase of Arabidopsis is not sufficient. Citrate supplementation in their experiments resulted in an increase in *n*-butanol production, an acetyl-CoA derived product, in the case of ATP-citrate lyase of *Arabidopsis*, which indicates that activity and affinity are issues for this enzyme in the yeast host. ATP-citrate lyase from an oleaginous yeast or carotenoid producing yeast like *Yarrowia lipolytica* or *Xanthophyllomyces dendrorhous* may be more promising as those organisms use ATP-citrate lyase as main acetyl-CoA source for the production of acetyl-CoA derived carotenoids and lipids [67, 68]. Indeed, other studies confirm that *in vivo* performance of heterologous enzymes needs to be optimized. Kozak *et al*. [44] functionally expressed a bacterial pyruvate dehydrogenase complex in *S*. *cerevisiae*. However, the addition of lipoate to growth media was required for activity and an increase in product formation of acetyl-CoA derived substances due to the bacterial complex has not yet been shown. In a further study, Kozak *et al*. [45] demonstrated that acetylating acetaldehyde dehydrogenase and pyruvate-formate lyase can replace acetyl-CoA synthetase of yeast but indicated that further research is essential for significant increases in the cytosolic acetyl-CoA supply. These studies confirm the necessity of further optimization of alternative reactions or pathways for a sufficient *in vivo* performance leading eventually to an increased product formation.

## Conclusions

Our previous *in silico* analysis [8] identified promising metabolic engineering targets, however, regulation and kinetics were not taken into account. This study demonstrates that regulation can interfere, such as observed with *KGD1* disruption, which had no effect in glucose-phase but in ethanol-phase as well as in glucose-limited conditions. Furthermore, *in vivo* activity of central carbon metabolism enzymes can interfere, such as demonstrated with ATP-citrate lyase or acetyl-CoA synthetase, resulting in poor *in vivo* performance. This necessity of further optimization was confirmed by other studies [44, 45]. Main sites of regulation need to be released and different sources for enzymes, rational protein design, or directed evolution might be essential to thoroughly fulfil desired activities of central carbon metabolism enzymes *in vivo* for an increased product formation from a heterologous host. These are issues to be addressed in the future.

Although the stoichiometric model was limited due to regulation and unpredicted enzyme activity, stoichiometric metabolic network analyses such as the one applied previously [8] can be used successfully as a metabolic flux prediction tool. Here, predictions were confirmed that ethanol is the more promising carbon source for sesquiterpenoid formation, and the metabolic flux in the strain carrying the α-ketoglutarate dehydrogenase disruption was redirected as predicted. Unfortunately, the intermediate acetate was produced instead of terpenoids. However, if the produced acetate could be efficiently converted to sesquiterpenoids, a titer in the g/L range could be achieved. This highlights that the *in silico* predicted strategy is indeed valuable and should be pursued in the future. Nevertheless, the inclusion of regulatory circuits and *in vivo* kinetics of enzymatic reactions into *in silico* metabolic network analyses are required to increase the predictive power of these models and thus reduce the time of strain improvement in the future.

## Supporting Information

S1 Fig Effect of dodecane on yeast cultures. Profiles of (a) biomass formation (cell dry weight, CDW), (b) glucose consumption, (c) ethanol formation, (d) glycerol formation and (e) acetate formation as a function of time as well as (f) ergosterol content of cells after glucose was exhausted. No second phase (○) or 7.5% dodecane (▲) were added to cultures of the yeast strain carrying pSP-GM1 grown in batch mode in shake flasks. Mean values and standard deviations of 3 experiments are shown.
(TIF)

S2 Fig Effectiveness of dodecane to capture patchoulol over time. 7.5% dodecane including patchoulol was added to sterile culture medium, flasks were incubated analogous to yeast strains and harvested. Patchoulol titer was determined at different time points. Mean values and standard deviations of 3 experiments are shown.
(TIF)

S3 Fig Profiles of patchoulol, glucose and biomass. Patchoulol formation (◇) was determined as a function of time together with biomass formation (cell dry weight, CDW) (▲) and glucose consumption (●) for the patchoulol producing yeast strain carrying pSP-P. Cells were grown in batch mode in shake flasks. Mean values and standard deviations of 3 experiments are shown.
(TIF)

S4 Fig Effect of t*HMG1* overexpression and fusion of FPP synthase and patchoulol synthase on physiological parameters of yeast. Profiles of physiological parameters of yeast strains carrying pSP-P (◇), pSP-Pt (●) and pSP-FPt (▲) grown in batch mode in shake flasks: (a) biomass formation (cell dry weight, CDW), (b) glucose consumption, (c) ethanol formation, (d) glycerol formation and (e) acetate formation as a function of time. Mean values and standard deviations of 3 experiments are shown.
(TIF)

S5 Fig Effect of ATP-citrate lyase overexpression on physiological parameters of yeast. Profiles of physiological parameters of yeast strains carrying pSP-FPt (○) and pSP-FPt-ACL (▲) in shake flask experiments: (a) biomass formation (cell dry weight, CDW) as a function of time, (b) glucose consumption, (c) ethanol formation, (d) glycerol formation and (e) acetate formation as a function of time. Mean values and standard deviations of 3 experiments are shown.
(TIF)

S6 Fig Effect of ATP-citrate lyase overexpression on relative fatty acid content. Shown is the relative fatty acid content of C12 to C18 of cells after 4 days of cultivation of yeast strains carrying pSP-FPt-ACL and pSP-FPt as control. Mean values and standard deviations of 3 experiments are shown.
(TIF)

S7 Fig Effect of citrate supplementation on sesquiterpenoid production. Sesquiterpenoid titer (in mg/L) after 4 days is shown for yeast strains carrying pSP-FPt and pSP-FPt-ACL in shake flask experiments without (C) and with 2.5 and 5 mM citrate added to the culture medium. Mean values and standard deviations of 3 experiments are shown.
(TIF)

S8 Fig Effect of disruption of *KGD1* on several parameters during glucose-limited conditions. (a) Cell growth (in generated CDW per flask), (b) acetate production, (c) sesquiterpenoids yield on glucose. Shown are mean values and standard deviations of three experiments. n.d., not detectable.
(TIF)

S9 Fig Effect of APT-citrate lyase overexpression on growth and sesquiterpenoid production during glucose-limited conditions. (a) Cell growth (in generated CDW per flask), (b) sesquiterpenoids yield on glucose. Shown are mean values and standard deviations of three experiments.
(TIF)

S10 Fig Plasmid maps of expression plasmids used in this study. (TIF)

S11 Fig Original Western Blot picture. (TIF)

S1 Table Gene sequences used in this study. (DOCX)

S2 Table Primers used in this study. (DOCX)

S3 Table Raw data of figures presented in this study. (XLSX)
