# Supplementary figures and images for: In Vivo Validation of In Silico Predicted Metabolic Engineering Strategies in Yeast: Disruption of α-Ketoglutarate Dehydrogenase and Expression of ATP-Citrate Lyase for Terpenoid Production
Source: PLoS One. 2015 Dec 23;10(12):e0144981. doi: 10.1371/journal.pone.0144981 (PMC4689373; doi:10.1371/journal.pone.0144981)

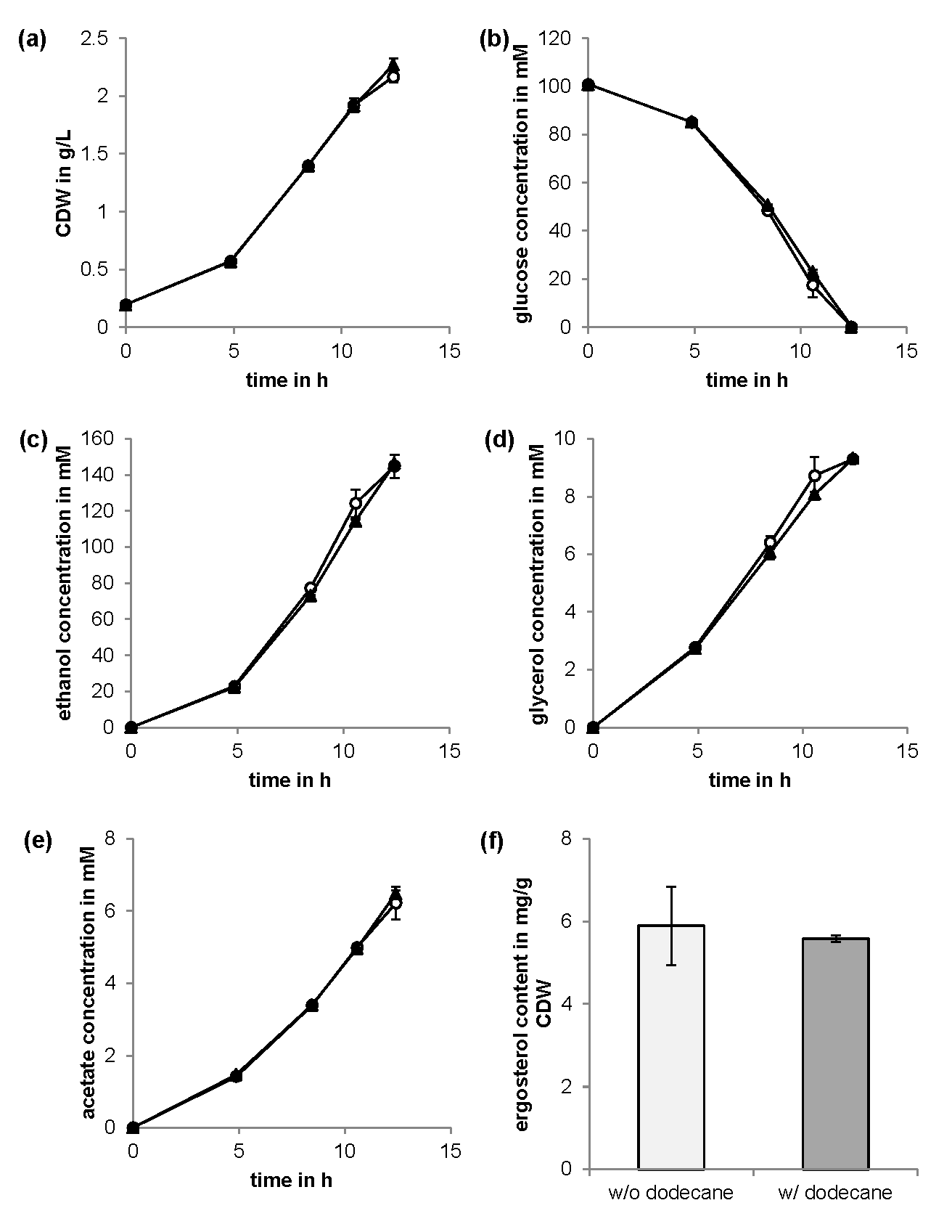

Supplement: S1 Fig — Profiles of (a) biomass formation (cell dry weight, CDW), (b) glucose consumption, (c) ethanol formation, (d) glycerol formation and (e) acetate formation as a function of time as well as (f) ergosterol content of cells after glucose was exhausted. No second phase (○) or 7.5% dodecane (▲) were added to cultures of the yeast strain carrying pSP-GM1 grown in batch mode in shake flasks. Mean values and standard deviations of 3 experiments are shown. (TIF) [file pone.0144981.s001.tif]

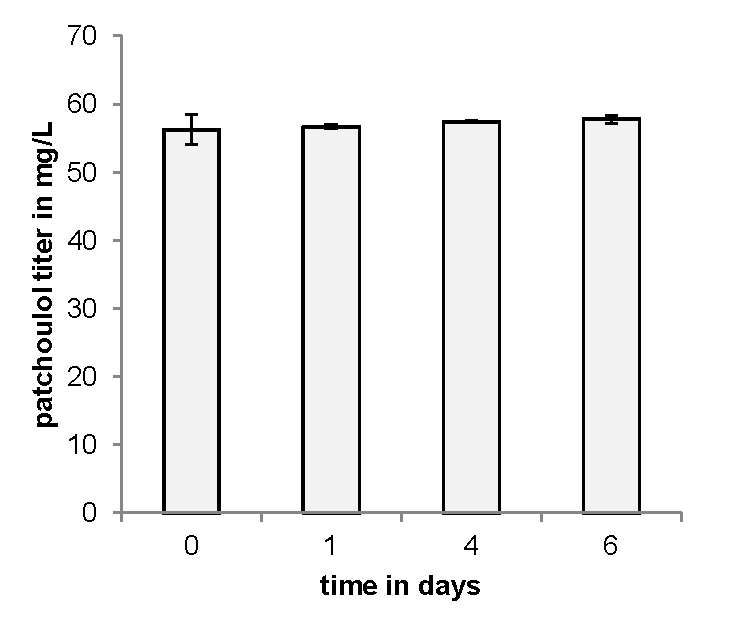

Supplement: S2 Fig — 7.5% dodecane including patchoulol was added to sterile culture medium, flasks were incubated analogous to yeast strains and harvested. Patchoulol titer was determined at different time points. Mean values and standard deviations of 3 experiments are shown. (TIF) [file pone.0144981.s002.tif]

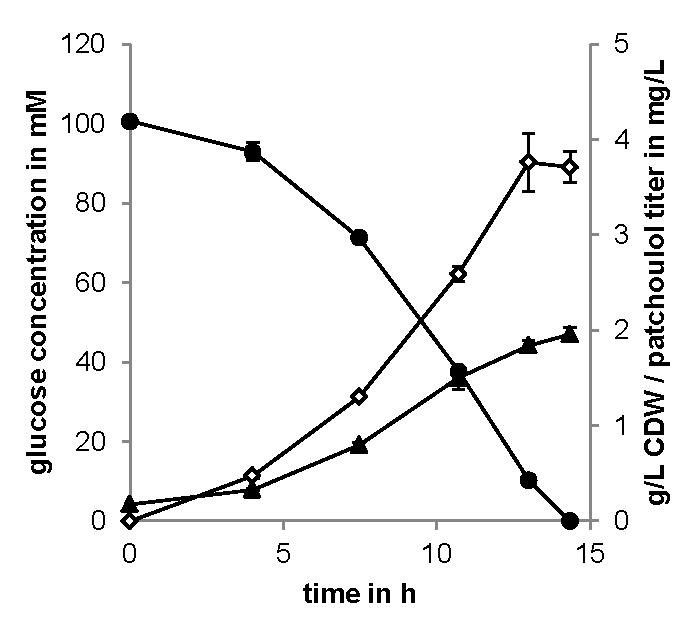

Supplement: S3 Fig — Patchoulol formation (◇) was determined as a function of time together with biomass formation (cell dry weight, CDW) (▲) and glucose consumption (●) for the patchoulol producing yeast strain carrying pSP-P. Cells were grown in batch mode in shake flasks. Mean values and standard deviations of 3 experiments are shown. (TIF) [file pone.0144981.s003.tif]

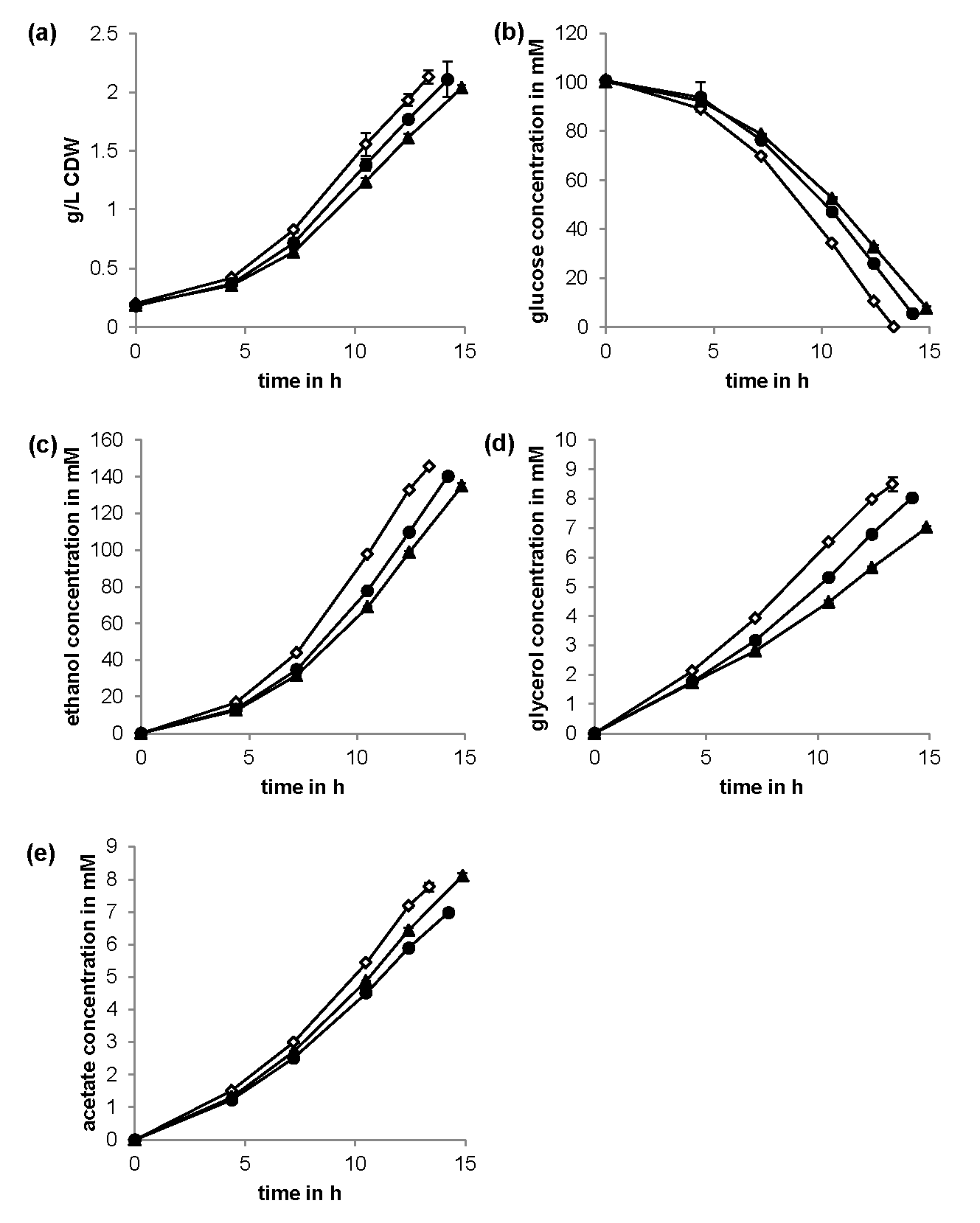

Supplement: S4 Fig — Profiles of physiological parameters of yeast strains carrying pSP-P (◇), pSP-Pt (●) and pSP-FPt (▲) grown in batch mode in shake flasks: (a) biomass formation (cell dry weight, CDW), (b) glucose consumption, (c) ethanol formation, (d) glycerol formation and (e) acetate formation as a function of time. Mean values and standard deviations of 3 experiments are shown. (TIF) [file pone.0144981.s004.tif]

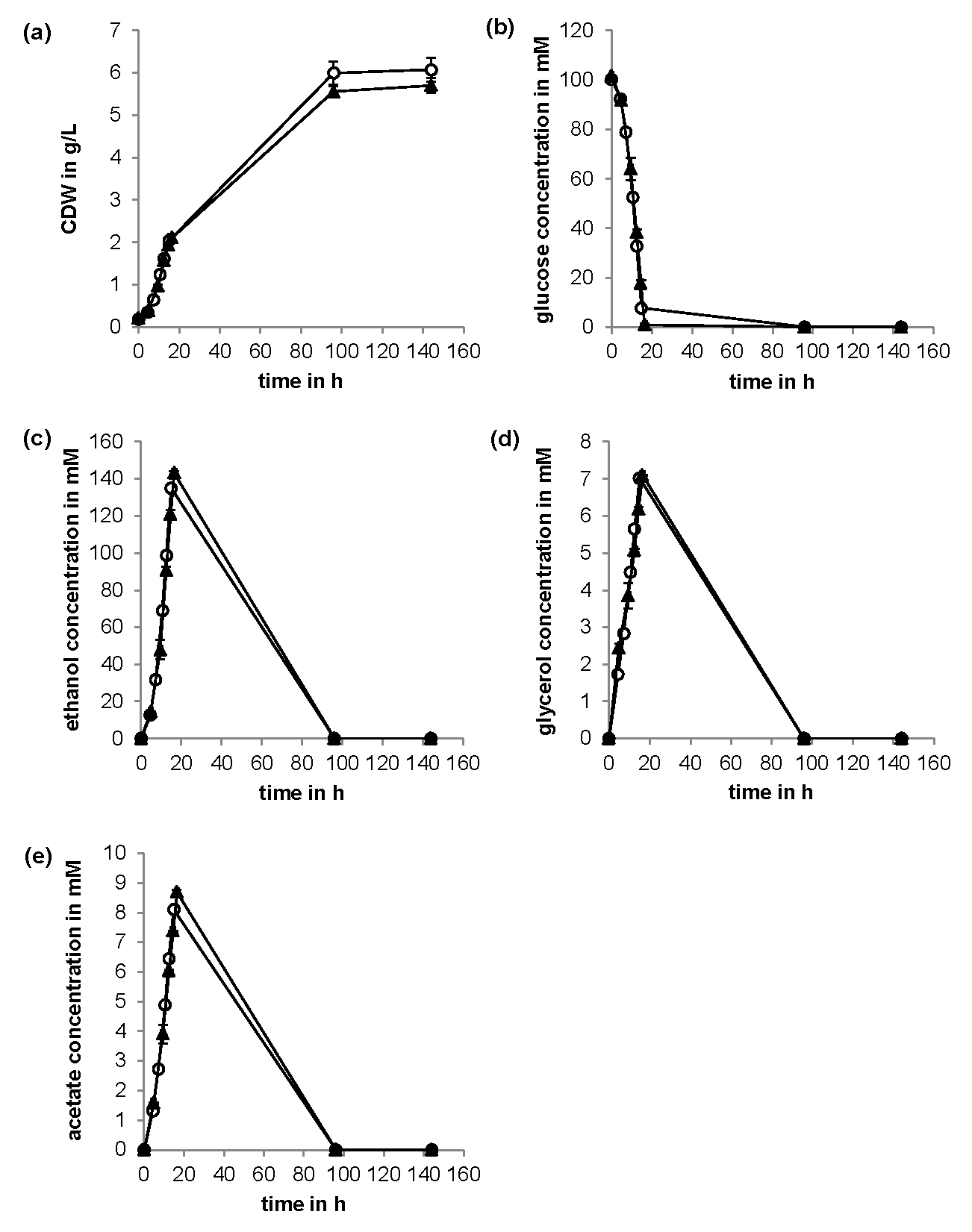

Supplement: S5 Fig — Profiles of physiological parameters of yeast strains carrying pSP-FPt (○) and pSP-FPt-ACL (▲) in shake flask experiments: (a) biomass formation (cell dry weight, CDW) as a function of time, (b) glucose consumption, (c) ethanol formation, (d) glycerol formation and (e) acetate formation as a function of time. Mean values and standard deviations of 3 experiments are shown. (TIF) [file pone.0144981.s005.tif]

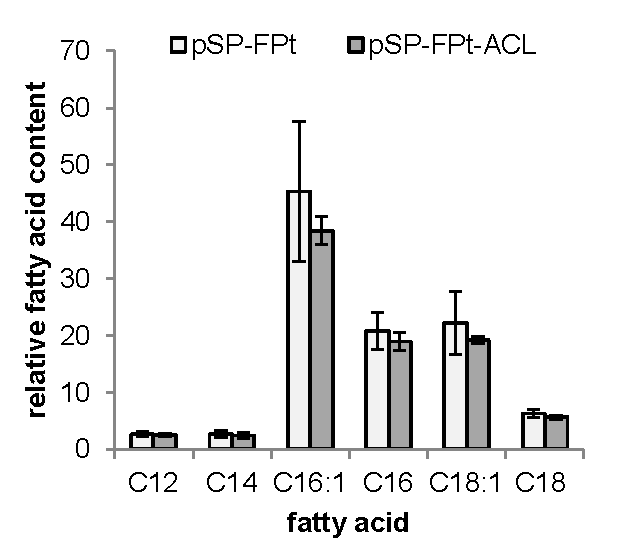

Supplement: S6 Fig — Shown is the relative fatty acid content of C12 to C18 of cells after 4 days of cultivation of yeast strains carrying pSP-FPt-ACL and pSP-FPt as control. Mean values and standard deviations of 3 experiments are shown. (TIF) [file pone.0144981.s006.tif]

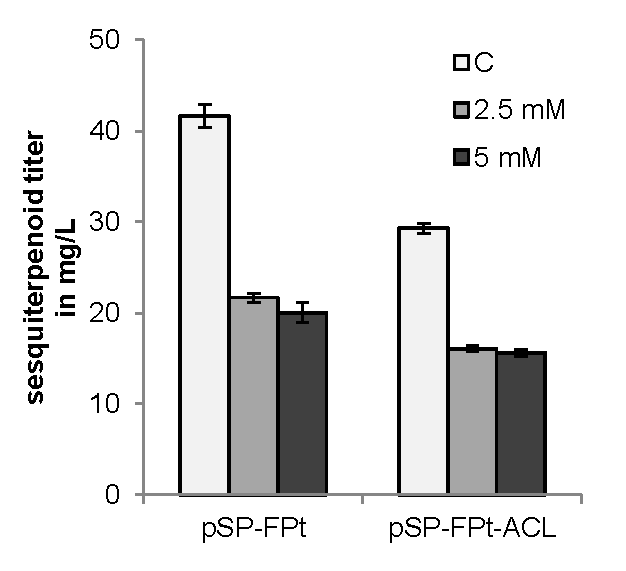

Supplement: S7 Fig — Sesquiterpenoid titer (in mg/L) after 4 days is shown for yeast strains carrying pSP-FPt and pSP-FPt-ACL in shake flask experiments without (C) and with 2.5 and 5 mM citrate added to the culture medium. Mean values and standard deviations of 3 experiments are shown. (TIF) [file pone.0144981.s007.tif]

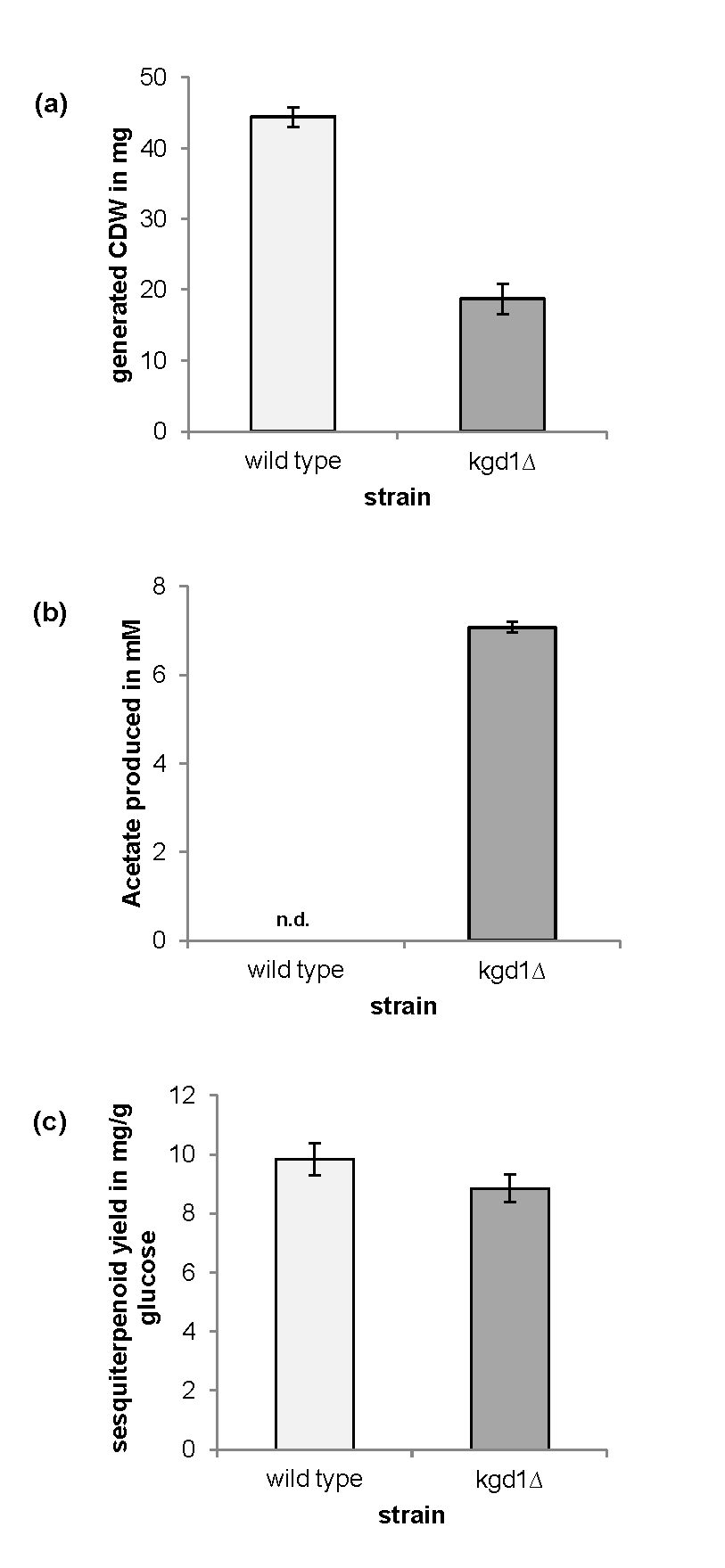

Supplement: S8 Fig — (a) Cell growth (in generated CDW per flask), (b) acetate production, (c) sesquiterpenoids yield on glucose. Shown are mean values and standard deviations of three experiments. n.d., not detectable. (TIF) [file pone.0144981.s008.tif]

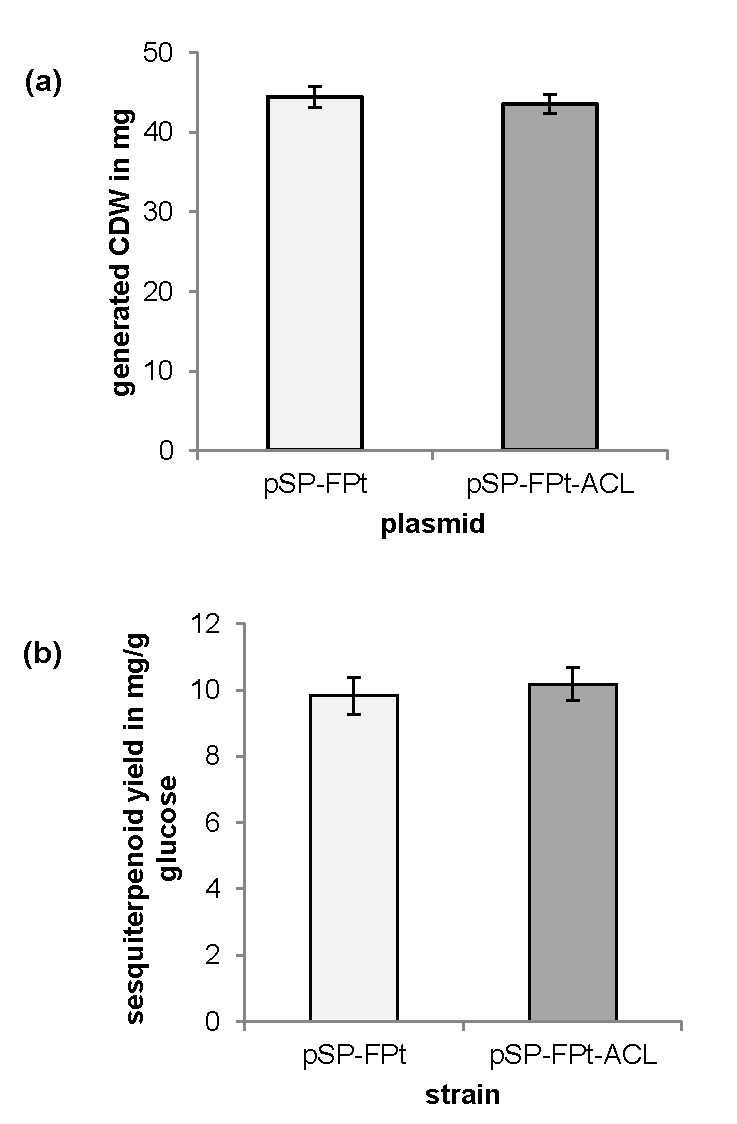

Supplement: S9 Fig — (a) Cell growth (in generated CDW per flask), (b) sesquiterpenoids yield on glucose. Shown are mean values and standard deviations of three experiments. (TIF) [file pone.0144981.s009.tif]

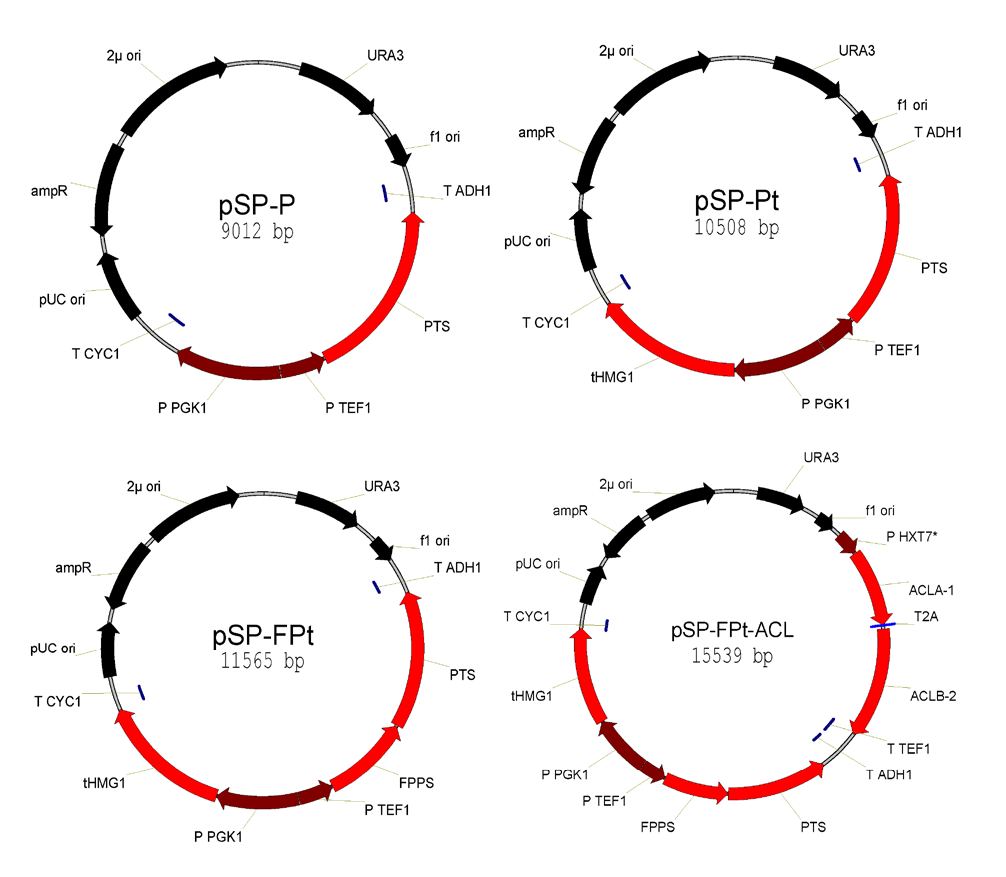

Supplement: S10 Fig — (TIF) [file pone.0144981.s010.tif]

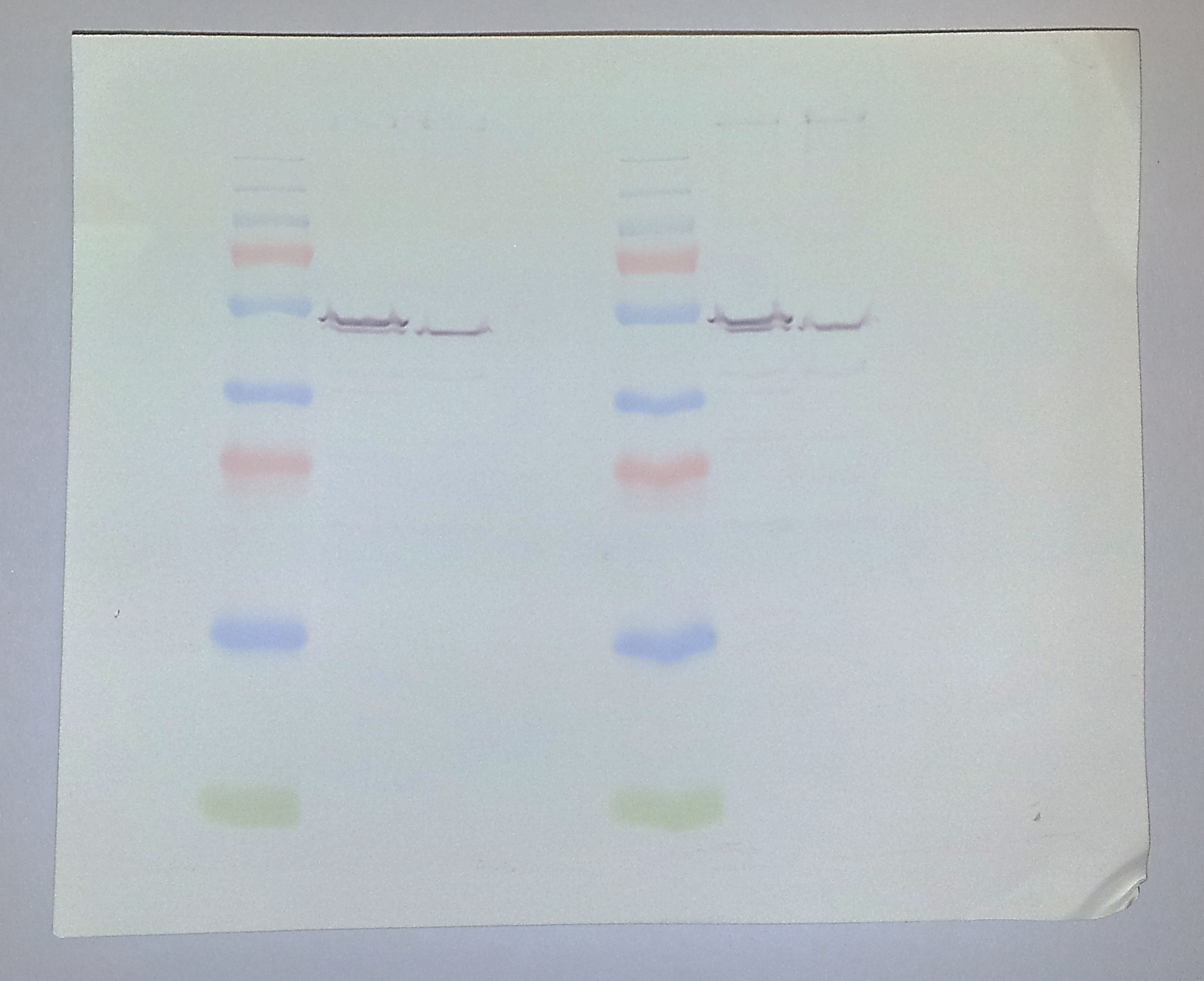

Supplement: S11 Fig — (TIF) [file pone.0144981.s011.tif]
