# Supplementary material for: In Vivo Validation of In Silico Predicted Metabolic Engineering Strategies in Yeast: Disruption of α-Ketoglutarate Dehydrogenase and Expression of ATP-Citrate Lyase for Terpenoid Production
Source: PLoS One. 2015 Dec 23;10(12):e0144981. doi: 10.1371/journal.pone.0144981 (PMC4689373; doi:10.1371/journal.pone.0144981)
Supplement: S1 Table — (DOCX) [file pone.0144981.s012.docx]

## Gene sequences used in this study.

| **Name** | **Sequence** |
| --- | --- |
| *PTS* | ATGGAATTATACGCCCAATCCGTTGGTGTTGGTGCTGCTTCTAGACCATTGGCTAATTTTCATCCATGTGTTTGGGGTGATAAGTTCATCGTTTACAACCCACAATCTTGTCAAGCTGGTGAAAGAGAAGAAGCTGAAGAATTGAAGGTCGAATTGAAGAGAGAATTGAAAGAAGCCTCCGACAACTACATGAGACAATTGAAAATGGTTGACGCCATCCAAAGATTGGGTATCGATTACTTGTTCGTTGAAGATGTTGATGAAGCCTTGAAGAACTTGTTCGAAATGTTCGATGCTTTCTGCAAGAACAACCATGATATGCATGCTACTGCTTTGTCCTTCAGATTATTGAGACAACACGGTTACAGAGTTTCTTGCGAAGTCTTTGAAAAGTTCAAGGATGGTAAGGACGGTTTCAAGGTTCCAAATGAAGATGGTGCTGTTGCTGTTTTGGAATTCTTTGAAGCTACCCATTTGAGAGTTCATGGTGAAGATGTATTGGATAACGCTTTCGATTTCACCAGAAACTACTTGGAATCTGTTTACGCTACTTTGAACGATCCAACTGCTAAGCAAGTTCATAATGCCTTGAACGAATTCTCATTCAGAAGAGGTTTGCCAAGAGTTGAAGCCAGAAAGTACATTTCCATCTACGAACAATATGCCTCCCATCATAAGGGTTTGTTGAAATTGGCTAAGTTGGACTTCAATTTGGTTCAAGCCTTGCACAGAAGAGAATTGTCTGAAGATTCTAGATGGTGGAAAACCTTGCAAGTTCCAACTAAGTTGTCCTTCGTTAGAGATAGATTGGTCGAATCTTACTTTTGGGCTTCTGGTTCTTACTTCGAACCTAATTATTCCGTTGCCAGAATGATTTTGGCTAAAGGTTTGGCTGTTTTGTCCTTGATGGATGATGTTTATGATGCCTACGGTACTTTCGAAGAATTGCAAATGTTCACCGATGCCATTGAAAGATGGGATGCTTCTTGTTTGGATAAGTTGCCAGATTACATGAAGATTGTCTACAAGGCTTTGTTGGACGTATTCGAAGAAGTTGACGAAGAATTGATTAAGTTGGGTGCTCCATATAGAGCCTACTATGGTAAAGAAGCTATGAAGTACGCTGCTAGAGCTTATATGGAAGAAGCTCAATGGAGAGAACAAAAGCACAAGCCAACTACCAAAGAATATATGAAGTTGGCTACTAAGACCTGCGGTTACATTACCTTGATTATCTTGTCATGCTTGGGTGTCGAAGAAGGTATCGTTACAAAAGAAGCTTTTGACTGGGTTTTCTCTAGACCACCTTTTATTGAAGCCACCTTGATCATTGCTAGATTGGTTAACGATATCACCGGTCACGAATTCGAAAAAAAGAGAGAACACGTTAGAACCGCTGTTGAATGTTACATGGAAGAACATAAGGTCGGTAAGCAAGAAGTTGTCTCCGAATTCTACAATCAAATGGAATCTGCTTGGAAGGACATCAACGAAGGTTTTTTAAGACCAGTCGAATTCCCAATCCCTTTGTTGTACTTGATCTTGAACTCTGTCAGAACCTTGGAAGTCATCTACAAAGAAGGTGATTCTTACACTCATGTTGGTCCAGCTATGCAAAACATTATCAAGCAATTATACTTGCACCCAGTTCCATATTAA |
| t*HMG* | ATGGACCAATTGGTGAAAACTGAAGTCACCAAGAAGTCTTTTACTGCTCCTGTACAAAAGGCTTCTACACCAGTTTTAACCAATAAAACAGTCATTTCTGGATCGAAAGTCAAAAGTTTATCATCTGCGCAATCGAGCTCATCAGGACCTTCATCATCTAGTGAGGAAGATGATTCCCGCGATATTGAAAGCTTGGATAAGAAAATACGTCCTTTAGAAGAATTAGAAGCATTATTAAGTAGTGGAAATACAAAACAATTGAAGAACAAAGAGGTCGCTGCCTTGGTTATTCACGGTAAGTTACCTTTGTACGCTTTGGAGAAAAAATTAGGTGATACTACGAGAGCGGTTGCGGTACGTAGGAAGGCTCTTTCAATTTTGGCAGAAGCTCCTGTATTAGCATCTGATCGTTTACCATATAAAAATTATGACTACGACCGCGTATTTGGCGCTTGTTGTGAAAATGTTATAGGTTACATGCCTTTGCCCGTTGGTGTTATAGGCCCCTTGGTTATCGATGGTACATCTTATCATATACCAATGGCAACTACAGAGGGTTGTTTGGTAGCTTCTGCCATGCGTGGCTGTAAGGCAATCAATGCTGGCGGTGGTGCAACAACTGTTTTAACTAAGGATGGTATGACAAGAGGCCCAGTAGTCCGTTTCCCAACTTTGAAAAGATCTGGTGCCTGTAAGATATGGTTAGACTCAGAAGAGGGACAAAACGCAATTAAAAAAGCTTTTAACTCTACATCAAGATTTGCACGTCTGCAACATATTCAAACTTGTCTAGCAGGAGATTTACTCTTCATGAGATTTAGAACAACTACTGGTGACGCAATGGGTATGAATATGATTTCTAAGGGTGTCGAATACTCATTAAAGCAAATGGTAGAAGAGTATGGCTGGGAAGATATGGAGGTTGTCTCCGTTTCTGGTAACTACTGTACCGACAAAAAACCAGCTGCCATCAACTGGATCGAAGGTCGTGGTAAGAGTGTCGTCGCAGAAGCTACTATTCCTGGTGATGTTGTCAGAAAAGTGTTAAAAAGTGATGTTTCCGCATTGGTTGAGTTGAACATTGCTAAGAATTTGGTTGGATCTGCAATGGCTGGGTCTGTTGGTGGATTTAACGCACATGCAGCTAATTTAGTGACAGCTGTTTTCTTGGCATTAGGACAAGATCCTGCACAAAATGTCGAAAGTTCCAACTGTATAACATTGATGAAAGAAGTGGACGGTGATTTGAGAATTTCCGTATCCATGCCATCCATCGAAGTAGGTACCATCGGTGGTGGTACTGTTCTAGAACCACAAGGTGCCATGTTGGACTTATTAGGTGTAAGAGGCCCACATGCTACCGCTCCTGGTACCAACGCACGTCAATTAGCAAGAATAGTTGCCTGTGCCGTCTTGGCAGGTGAATTATCCTTATGTGCTGCCCTAGCAGCCGGCCATTTGGTTCAAAGTCATATGACCCACAACAGGAAACCTGCTGAACCAACAAAACCTAACAATTTGGACGCCACTGATATAAATCGTTTGAAAGATGGGTCCGTCACCTGCATTAAATCCTAA |
| FPP synthase: *FPPS* | ATGGCTTCAGAAAAAGAAATTAGGAGAGAGAGATTCTTGAACGTTTTCCCTAAATTAGTAGAGGAATTGAACGCATCGCTTTTGGCTTACGGTATGCCTAAGGAAGCATGTGACTGGTATGCCCACTCATTGAACTACAACACTCCAGGCGGTAAGTTAAATAGAGGTTTGTCCGTTGTGGACACGTATGCTATTCTCTCCAACAAGACCGTTGAACAATTGGGGCAAGAAGAATACGAAAAGGTTGCTATTCTAGGTTGGTGCATTGAGTTGTTGCAGGCTTACTTCTTGGTCGCCGATGATATGATGGACAAGTCCATTACCAGAAGAGGCCAACCATGTTGGTACAAGGTTCCTGAAGTTGGGGAAATTGCCATCAATGACGCATTCATGTTAGAGGCTGCTATCTACAAGCTTTTGAAATCTCACTTCAGAAACGAAAAATACTACATAGATATCACCGAATTGTTCCATGAAGTCACCTTCCAAACCGAATTGGGCCAATTGATGGACTTAATCACTGCACCTGAAGACAAAGTCGACTTGAGTAAGTTCTCCCTAAAGAAGCACTCCTTCATAGTTACTTTCAAGACTGCTTACTATTCTTTCTACTTGCCTGTCGCATTGGCTATGTACGTTGCCGGTATCACAGATGAAAAGGATTTGAAACAAGCCAGAGATGTCTTGATTCCATTGGGTGAATATTTCCAAATTCAAGATGACTACTTAGACTGCTTCGGTACCCCAGAACAGATCGGTAAGATCGGTACAGATATCCAAGATAACAAATGTTCTTGGGTAATCAACAAGGCATTAGAACTTGCTTCCGCAGAACAAAGAAAGACTTTAGACGAAAATTACGGTAAGAAGGACTCAGTCGCAGAAGCCAAATGCAAAAAGATTTTCAATGACTTGAAAATCGACCAGTTATACCACGAATATGAAGAGTCTGTTGCCAAGGATTTGAAGGCCAAGATCTCCCAAGTCGACGAGTCTCGTGGCTTCAAAGCCGACGTCTTAACTGCGTTTTTGAACAAAGTTTACAAGAGAAGCAAA |
| *ACLA-1_T2A_ACLB-2* | ATGGCGAGGAAGAAGATCAGAGAGTATGACTCAAAGAGGTTGGTGAAGGAACATTTCAAAAGGCTTTCTGGCAAAGAGCTTCCTATCAGATCCGTTCAGGCAGATGATGTTTTCTGGTTACATTCTTGCTTTGGTTTCTCCGACATTAGGCACCATGTGATTAATGAAACAACTGATCTAAATGAGCTAGTTGAAAAGGAACCTTGGCTCTCGTCTGAGAAGCTGGTGGTGAAACCTGACATGTTGTTTGGAAAGCGTGGCAAGAGTGGTTTGGTTGCCTTGAAATTAGATTTTGCTGATGTTGCCACTTTTGTTAAAGAACGTTTGGGAAAAGAGGTAGAGATGAGTGGATGCAAAGGACCCATAACAACATTCATAGTTGAACCATTTGTTCCACACAATGAGGAGTATTATCTCAATGTTGTCTCGGATCGGCTTGGTTGCAGCATAAGCTTTTCTGAGTGTGGAGGAATTGAGATCGAGGAGAACTGGGACAAGGTCAAGACAATATTTTTACCAACAGGTGCTTCCCTGACACCTGAAATATGTGCACCTCTTGTCGCAACTCTTCCCTTAGAGATCAAAGCTGAAATTGAAGAATTTATCAAAGTCATTTTCACCCTATTCCAAGATCTTGATTTCACTTTCTTGGAGATGAATCCTTTCACTCTAGTTGATGGAAGTCCTTATCCTCTGGATATGAGGGGTGAGCTTGATGATACTGCTGCCTTCAAAAACTTTAAAAAATGGGGCGACATTGAATTTCCTCTGCCATTTGGAAGAGTAATGAGTCCTACAGAAAGCTTTATCCACGGACTGGATGAGAAGACAAGTGCGTCTTTGAAGTTTACCGTTCTGAACCCCAAGGGACGGATTTGGACAATGGTAGCTGGTGGAGGAGCAAGTGTCATCTATGCGGATACGGTTGGAGATCTCGGGTATGCATCTGAACTTGGCAACTATGCTGAATACAGTGGAGCACCCAAAGAAGATGAGGTTTTGCAGTACGCCAGAGTCGTTATTGATTGTGCTACAGCAAACCCGGATGGAAAAAGCAGAGCCCTTGTCATCGGAGGCGGAATTGCCAACTTCACTGACGTTGCTGCTACTTTCAATGGCATAATCCGCGCTCTTAAAGAAAAGGAAGCAAAGCTGAAAGCAGCAAGGATGCATATATTTGTGAGGAGAGGAGGACCAAACTACCAAAAGGGACTTGCTAAAATGCGAGCCCTTGGAGATGATATCGGTGTCCCCATCGAGGTCTATGGCCCAGAAGCAACCATGACAGGTATCTGCAAGGAGGCAATCCAGTACATCACAGCAGCAGCA**AGAGCAGAAGGAAGGGGTTCT**TTG**TTGACTTGTGGAGATGTTGAGGAGAATCCAGGACCA**GCAACGGGACAGCTTTTTTCTCGTACCACACAAGCTTTGTTCTACAACTATAAGCAGCTTCCTGTTCAACGAATGCTCGATTTCGACTTTCTCTGTGGACGTGAAACGCCTTCTGTTGCTGGAATCATAAATCCTGGTTCTGAAGGTTTTCAAAAGCTCTTTTTCGGGCAGGAGGAAATCGCTATCCCTGTTCATGCCGCCATTGAGGCAGCTTGTGCTGCGCATCCAACAGCGGATGTATTCATCAACTTTGCATCTTTTAGGAGTGCTGCTGCTTCATCCATGGCTGCTTTGAAGCAGCCGACTATTAAAGTTGTGGCAATTATAGCTGAAGGTGTTCCAGAATCAGACACTAAGCAGCTGATTGCGTATGCTCGTGCAAACAATAAGGTTGTTATTGGACCGGCTACTGTTGGAGGTATTCAAGCTGGAGCCTTTAAGATTGGTGATACTGCAGGAACAATTGATAACATTATCCAGTGCAAGCTATACAGACCTGGATCTGTTGGTTTTGTCTCCAAATCTGGTGGAATGTCTAATGAAATGTACAATACTGTTGCCCGTGTGACTGATGGGATCTACGAAGGCATTGCTATTGGTGGAGACGTGTTCCCAGGATCGACTTTATCTGACCACATCCTTCGGTTTAACAACATCCCACAGATAAAAATGATGGTTGTACTTGGAGAGCTTGGAGGAAGAGATGAATACTCTCTTGTTGAAGCTTTGAAAGAGGGAAAAGTCAATAAACCTGTGGTTGCTTGGGTCAGTGGAACTTGTGCACGACTCTTCAAGTCTGAAGTACAGTTTGGTCATGCAGGTGCCAAAAGTGGCGGCGAGATGGAGTCTGCACAAGCCAAGAATCAAGCTCTCATAGATGCTGGAGCTATTGTTCCCACTTCATTTGAAGCTCTAGAATCTGCAATCAAAGAGACTTTTGAGAAACTGGTTGAAGAAGGAAAGGTCTCTCCTATCAAGGAAGTCATTCCTCCACAAATCCCTGAGGATCTCAATTCTGCAATTAAGAGTGGGAAAGTCCGGGCTCCTACTCACATCATCTCCACCATATCTGATGACAGAGGGGAGGAACCATGCTATGCTGGTGTTCCAATGTCTTCCATCATCGAACAAGGCTATGGAGTGGGTGATGTCATTTCCCTTCTATGGTTCAAACGTAGTCTACCTCGTTACTGTACAAAATTCATTGAGATATGCATAATGCTGTGTGCTGATCACGGTCCATGCGTCTCCGGCGCTCACAACACCATTGTAACAGCAAGAGCAGGCAAAGACCTCGTCTCAAGTCTTGTCTCAGGTTTATTGACCATTGGTCCCCGATTTGGTGGTGCCATTGATGACGCTGCTCGATACTTCAAAGACGCGTGTGACAGGAATCTCACACCTTATGAATTTGTTGAGGGAATGAAGAAAAAGGGAATCCGAGTCCCCGGGATTGGACACAGGATCAAGAGCAGAGACAACAGAGACAAAAGAGTGGAGCTTCTTCAGAAATTTGCTCGGTCCAACTTCCCATCAGTGAAGTACATGGAGTACGCAGTGACAGTGGAGACATACACGCTCTCAAAGGCAAACAACCTCGTACTCAACGTTGATGGAGCCATTGGATCTCTCTTCTTGGACCTTCTAGCTGGAAGTGGGATGTTCACTAAACAAGAGATTGACGAGATTGTTCAGATCGGTTATCTCAACGGTCTGTTTGTTCTTGCTCGCTCCATCGGTTTGATCGGGCACACGTTTGATCAGAAGAGATTGAAGCAGCCACTGTATCGTCACCCATGGGAAGATGTGTTGTACACCAAGTAA |

T2A sequence from *Thosea asigna* virus is marked in bold.
