# Supplementary material for: In Vivo Validation of In Silico Predicted Metabolic Engineering Strategies in Yeast: Disruption of α-Ketoglutarate Dehydrogenase and Expression of ATP-Citrate Lyase for Terpenoid Production
Source: PLoS One. 2015 Dec 23;10(12):e0144981. doi: 10.1371/journal.pone.0144981 (PMC4689373; doi:10.1371/journal.pone.0144981)
Supplement: S2 Table — (DOCX) [file pone.0144981.s013.docx]

## Primers used in this study.

| **Name** | **Sequence (5’-3’)** |
| --- | --- |
| MF0018 | **GCAATCTAATCTAAGTTTTAATTACAA**GCGGCCGCAACAAAATGGAATTATACG |
| MF0019 | **CTTGACCAAACCTCTGGCGAAGAATTG**TTAATTAATTAATATGGAACTGGGTGC |
| MF0016_S | CCATCTTTTCGTAAATTTC |
| MF0017_S | CATTTTTCTTGTTCTATTACAAC |
| EG_tHMG_Fw | **GTAATTATCTACTTTTTACAACAAATATAAAACAA**AAAACAATGGACCAATTGGTGAAAACTG |
| EG_tHMG_Rv | **CTAACTCCTTCCTTTTCGGTTAGAGCGGATCTTA**TTAGGATTTAATGCAGGTG |
| MF0014_S | ACAGATCATCAAGGAAG |
| MF0015_S | GTTACATGCGTACACGC |
| EMG_FPPS_Fw | **CATAGCAATCTAATCTAAGTTTTAATTACAA**AACAAAATGGCTTCAGAAAAAGAAATTAG |
| EMG_FPPS_Rv | **CAACACCAACGGATTGGGCGTATAATTCCAT**ACCAGAACCTTTGCTTCTCTTGTAAACTTTG |
| EMG_FPPS_S | CATGGATGAAAATTAGCC |
| EMG_ACL_1_pSP+ PHXT7 | **CGATCGGTGCGGGCCTCTTCGCTATTACGCCAGCTGGATAAAGGCGCGCC**TACGCCAAGCGCGCAATTAAC |
| EMG_ACL_2_PHXT7+ACLA-1 | **CTTCCTCGCCATTGTT**TTTTTGATTAAAATTAAAAAAACTTTTTGTTTTTG |
| EMG_ACL_3_PHXT7+ACLA-1 | **CAAAGAATAAACACAAAAACAAAAAGTTTTTTTAATTTTAATCAAAAAAACA**ATGGCGAGGAAGAAGATCAG |
| EMG_ACL_4_ACLA-1+T2A* | **TGGTCCTGGATTCTCCTCAACATCTCCACAAGTCAACAAAGAACCCCTTCCTTCTGCTCT**TGCTGCTGCTGTGATGTACTG |
| EMG_ACL_5_T2A+ACLB-2* | **AGAGCAGAAGGAAGGGGTTCTTTGTTGACTTGTGGAGATGTTGAGGAGAATCCAGGACCA**GCAACGGGACAGCTTTTTTC |
| EMG_ACL_6_ACLB-2+TTEF | **CAAATGACAAGTTCTTGAAAACAAGAATCTTTTTATTGTCAGTACTGA**TTACTTGGTGTACAACACATCTTCCC |
| EMG_ACL_7_ACLB-2+TTEF | **CACCAAGTAA**TCAGTACTGACAATAAAAAGATTC |
| EMG_ACL_8_TTEF+AscI+pSP | **CTCAGGTATAGCATGAGGTCGCTCCAATTCCTAGGTCGTTTGGCGCGCC**AATACGACTCACTATAGGGAG |
| EMG_ACL_9_S | GAACGTGGCGAGAAAGGAA |
| EMG_ACL_10_S | TCTTTGGGTGCTCCACTGT |
| EMG_ACL_11_S | GGGCGACATTGAATTTCCT |
| EMG_ACL_12_S | GGGATTTGTGGAGGAATGA |
| EMG_ACL_13_S | AGTTTGGTCATGCAGGTGCC |
| EMG_ACL_14_S | TGTCGGCTTGTCTACCTTGC |
| EMG_KGD_Fw | **CCCCAAATTAAAGTTTCGTTTGAAAGAAACAACAAAAGAGAAAGAAAGC**TTCGTACGCTGCAGGTCGAC |
| EMG_KGD_Rv | **GATGGGACGTAATCCTTTGCCTTTTCGAAAGCATCTTCAAATAAGTTCC**GCATAGGCCACTAGTGGATCTG |
| EMG_KGD_S1 | TCTCGTTCAGCATCATACT |
| EMG_KGD_S2 | GCAGCAGTTAACCATTCT |

Flanking homologous regions are marked in bold, asterisk (*) indicates PAGE purified primers.
